# Heterogeneity in design and analysis of ICU delirium randomized trials: a systematic review

**DOI:** 10.1186/s13063-021-05299-1

**Published:** 2021-05-20

**Authors:** Elizabeth Colantuoni, Mounica Koneru, Narjes Akhlaghi, Ximin Li, Mohamed D. Hashem, Victor D. Dinglas, Karin J. Neufeld, Michael O. Harhay, Dale M. Needham

**Affiliations:** 1 Department of Biostatistics, Bloomberg School of Public Health, Johns Hopkins University, Baltimore, MD USA; 2 Outcomes After Critical Illness and Surgery, Johns Hopkins School of Medicine, Baltimore, MD USA; 3 Pulmonary and Critical Care Medicine, Department of Medicine, Johns Hopkins University School of Medicine, Baltimore, MD USA; 4 Department of Medicine, Marshfield Clinic, Marshfield, WI USA; 5 Department of Psychiatry and Behavioral Sciences, Johns Hopkins University, School of Medicine, Baltimore, MD USA; 6 Department of Epidemiology, Perelman School of Medicine, University of Pennsylvania, Philadelphia, PA USA; 7 Department of Medicine, Perelman School of Medicine, University of Pennsylvania, Philadelphia, PA USA; 8 PAIR (Palliative and Advanced Illness Research) Center Clinical Trials Methods and Outcomes Lab, Perelman School of Medicine, University of Pennsylvania, Philadelphia, PA USA; 9 Physical Medicine and Rehabilitation, Johns Hopkins University School of Medicine, Baltimore, MD USA

**Keywords:** Systematic review, Randomized trials, Critically ill patients, Delirium, Outcome definition, Statistical methods

## Abstract

**Background:**

There is a growing number of randomized controlled trials (RCTs) evaluating interventions to prevent or treat delirium in the intensive care unit (ICU). Efforts to improve the conduct of delirium RCTs are underway, but none address issues related to statistical analysis. The purpose of this review is to evaluate heterogeneity in the design and analysis of delirium outcomes and advance methodological recommendations for delirium RCTs in the ICU.

**Methods:**

Relevant databases, including PubMed and Embase, were searched with no restrictions on language or publication date; the search was conducted on July 8, 2019. RCTs conducted on adult ICU patients with delirium as the primary outcome were included where trial results were available. Data on frequency and duration of delirium assessments, delirium outcome definitions, and statistical methods were independently extracted in duplicate. The review was registered with PROSPERO (CRD42020141204).

**Results:**

Among 65 eligible RCTs, 44 (68%) targeted the prevention of delirium. The duration of follow-up varied, with 31 (48%) RCTs having 7 days of follow-up, and only 24 (37%) conducting delirium assessments after ICU discharge. The incidence of delirium was the most common outcome (50 RCTs, 77%) for which 8 unique statistical methods were applied. The most common method, applied to 51 of 56 (91%) delirium incidence outcomes, was the two-sample test comparing the proportion of patients who ever experienced delirium. In the presence of censoring of patients at ICU discharge or death, this test may be misleading. The impact of censoring was also not considered in most analyses of the duration of delirium, as evaluated in 24 RCTs, with 21 (88%) delirium duration outcomes analyzed using a non-parametric test or two-sample *t* test. Composite outcomes (e.g., rank-based delirium- and coma-free days), used in 11 (17%) RCTs, seldom explicitly defined how ICU discharge, and death were incorporated into the definition and were analyzed using non-parametric tests (11 of 13 (85%) composite outcomes).

**Conclusions:**

To improve delirium RCTs, outcomes should be explicitly defined. To account for censoring due to ICU discharge or death, survival analysis methods should be considered for delirium incidence and duration outcomes; non-parametric tests are recommended for rank-based delirium composite outcomes.

**Trial registration:**

PROSPERO CRD42020141204. Registration date: 7/3/2019.

**Supplementary Information:**

The online version contains supplementary material available at 10.1186/s13063-021-05299-1.

## Background

Delirium is a clinical syndrome in which patients have fluctuating impairments in attention and cognition [1]. This syndrome is highly prevalent among patients in the intensive care unit (ICU), with prevalence ranging from 50 to 80% [2, 3]. Delirium is associated with longer durations of mechanical ventilation and ICU stay, as well as increased risk of mortality [4]. Moreover, delirium is associated with long-term cognitive impairments [5, 6].

The number of randomized controlled trials (RCTs) evaluating interventions to prevent or treat delirium in ICU patients has been increasing. There are ongoing efforts to establish standards for conducting such RCTs [7, 8]. Moreover, there are efforts to establish a core set of outcomes and associated measurement instruments for delirium RCTs in the ICU (https://deliriumnetwork.org/measurement/#measurement-resources, [9]) given important heterogeneity in these areas among existing RCTs [10].

The development of a core set of outcomes and measurement instruments are key steps towards improving comparability and harmonization across delirium RCTs. As highlighted by an international interprofessional panel [8], improving the conduct of delirium RCTs also requires evaluating heterogeneity in the statistical methods applied to delirium outcomes. Standardization of statistical methods will allow for improved comparison of the effect of interventions while appropriately accounting for key features of RCT design and patient population [11,16]. Hence, to advance the understanding of RCT design and statistical analysis of delirium outcomes and to assist with advancing methodologic recommendations, we undertook a systematic review of published delirium RCTs and provide related recommendations for the field.

## Methods

This systematic review was funded by the U.S. National Institutes of Health (R01AG061384), registered with PROSPERO (CRD42020141204) and reported in accordance with the PRISMA guideline ([17], Additional File Section 1).

### Search strategy and selection criteria

An experienced medical librarian participated in designing the literature search strategy, which was peer-reviewed by another medical librarian prior to use. We searched the following databases: PubMed, Cochrane Library, CINAHL, Embase, Scopus, Web of Science, PsycINFO, and ClinicalTrials.gov. The search strategy was designed around the following key search terms: critical illness, delirium, and randomized trial (Additional File Section 2). There were no restrictions on language or publication date. The search was conducted on July 8, 2019.

The title and abstract of identified citations were independently screened, in duplicate, followed by independent, duplicate screening of the full text of the citations by trained research staff. The first author (EC) adjudicated discrepancies between these reviewers. Citations were included if they were the primary publication of a RCT of any intervention(s) (with any type of control group) individually randomized to patients treated in an ICU, with the primary outcome being delirium evaluated using a validated screening instrument ([18], Additional File Section 3) or diagnostic criteria [1]. In addition, we conducted hand searches of references from eligible citations, of three recent systematic reviews [19,21], and the Network for Investigating Delirium: Unifying Scientists (NIDUS) registry of delirium studies (https://deliriumnetwork.org/delirium-research-hub/).

### Data extraction and quality assessment

The final data elements for extraction, and associated REDCap database, were derived after three rounds of iterative pilot testing. Data elements were extracted independently, in duplicate, with discrepancies resolved via consensus among the data extractors. Key RCT characteristics were extracted, including trial type (prevention only, treatment only, or both prevention and treatment), sample size, funding source, country, patient population, ICU type, and patient characteristics. Data on the delirium screening or diagnostic instrument, delirium assessment frequency, and duration of assessment were extracted. Delirium outcomes, a priori classified into four categories (Additional File Section 4) were recorded, as was outcome type (primary, secondary, or reported but not named as a primary or secondary outcome), and the statistical method(s) applied (Additional File Section 5). Similar data were collected for patient mortality and ICU length of stay. We recorded whether an analysis of the primary delirium outcome included adjustment for baseline variables, regardless of whether this analysis was the primary analysis or a secondary analysis. Study results for all delirium outcomes, mortality, and ICU length of stay were reported. The risk of bias was independently assessed by two raters, using the Cochrane Risk of Bias Tool [22]. Study team members with training in epidemiology (MDH, DN, MOH) or biostatistics (XL, EC) extracted all data elements related to delirium outcome definition(s) and statistical analysis methods, in addition to completing the risk of bias assessment.

### Data synthesis and analysis

The data were evaluated to identify potential outliers and missing values. All missing values were reviewed by study team members (MK, NA, and EC) and resolved when possible using a full-text review or contacting corresponding authors. Delirium outcomes and statistical methods were categorized by two biostatisticians with masters or doctoral training in biostatistics (EC and XL). Summary statistics of extracted data were computed for all studies and by trial type. In addition, recognizing key differences in surgery (cardiac and general surgery) and critically ill patients, i.e., mechanically ventilated, acute respiratory failure, or acute respiratory distress syndrome (MV, ARF, ARDS) patients, the statistical methods applied to delirium outcomes were summarized separately for RCTs conducted among surgery (cardiac and general surgery) vs. critically ill patients.

## Results

### Study characteristics

**Fig. 1:**
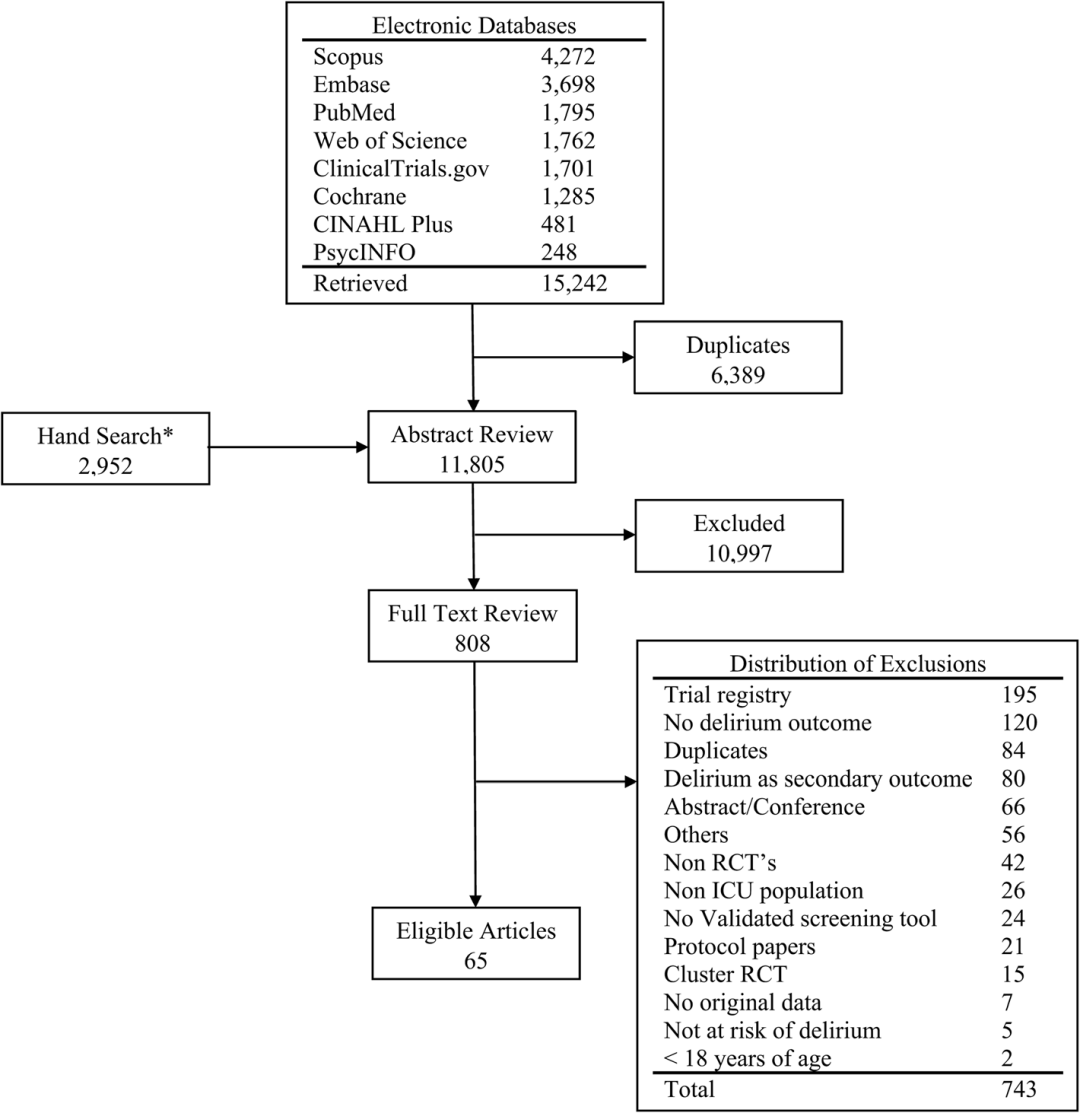
Literature Search Flow Chart. *We hand searched all the references of the eligible articles, the NIDUS bibliography (https://deliriumnetwork.org/delirium-research-hub/), and articles from relevant systematic reviews [19,21] and compared to the deduplicated articles from the electronic database search

The comprehensive search strategy identified 15,242 citations. After removing duplicates, we reviewed the title and abstract of 11,805 citations and subsequently completed the full-text review of 808 citations. We identified 65 delirium RCTs, published between 2003 and 2019 (quartiles: 2003, 2016, 2017), that met the inclusion criteria (Fig. 1 and Table 1). Of these, 44 (68%), 12 (18%), and 9 (14%) focused on delirium prevention only, treatment only, or both prevention and treatment, respectively (Table 1). The majority of the 65 RCTs were two-arm trials (a single intervention with the control group, *n*= 54, 83%) with 9 (14%) multi-arm and 2 (3%) factorial trials. The RCTs, including 12 foreign language papers (9 Chinese, 1 Italian, 1 Persian, and 1 Turkish), were conducted predominantly in the USA (*n*=16, 25%), China (12, 18%), and Iran (8, 12%), with only 20 (31%) reporting government-funding. Two members of the study team members (XL and NA) who were native speakers reviewed the Chinese and Persian articles. We reached out to bilingual collaborators who have expertise in delirium/research to help with the Italian and Turkish articles. The three most common patient populations were cardiac surgery (22, 34%), surgery (19, 29%), and ARF (17, 26%) patients. Among the 65 RCTs, the median (interquartile range) of the average patient age was 62 (59, 69) years old, and the median proportion of males was 62% (53%, 72%).

**Table 1:** Study and patient characteristics for the 65 delirium trials

	Overall	Prevention	Treatment	Both
	***n*** = 65	***n*** = 44	***n*** = 12	***n*** = 9
**Study characteristics**				
Sample size, median [Q1,Q3]	113 [70, 198]	109 [70, 158]	104 [72, 238]	141[101, 164]
Study start year, median [Q1, Q3]	2012 [2009, 2014]	2013 [2009, 2014]	2009 [2005, 2012]	2010 [2009, 2011]
Funding^a^				
Governmental funding	20 (31)	9 (21)	5 (42)	6 (67)
No external funding	18 (28)	12 (27)	3 (25)	3 (33)
Industry funding	8 (12)	5 (11)	2 (17)	1 (11)
Non-profit sources (non-govt)	5 (8)	3 (7)	2 (17)	0 (0)
Other source of funding	7 (11)	6 (14)	0 (0)	1 (11)
Unclear funding source	16 (25)	13 (30)	3 (25)	0 (0)
Country/region^a^				
USA	16 (25)	10 (23)	4 (33)	2 (22)
China	12 (19)	10 (23)	1 (8)	1 (11)
Iran	8 (12)	7 (16)	1 (8)	0 (0)
Canada	5 (8)	4 (9)	1 (8)	0 (0)
Europe^b^	5 (8)	2 (5)	0 (0)	3 (33)
Japan	2 (3)	2 (5)	0 (0)	0 (0)
Other^c^	21 (32)	12 (27)	5 (42)	3 (33)
Patient type^a,e^				
Cardiac surgery	22 (34)	16 (36)	3 (25)	3 (33)
Surgery	19 (29)	15 (34)	3 (325)	1 (11)
ARF^f^	17 (26)	10 (23)	3 (25)	4 (44)
Medical	6 (9)	2 (5)	3 (25)	1 (11)
Other	14 (22)	6 (14)	6 (50)	2 (22)
ICU type^a^				
General or mixed^d^	13 (20)	6 (14)	4 (33)	3 (33)
Surgical	11 (17)	6 (14)	4 (33)	1 (11)
Cardiovascular surgery	10 (15)	10 (23)	0 (0)	0 (0)
Medical	9 (14)	4 (9)	4 (33)	1 (11)
Coronary care	1 (2)	0 (0)	0 (0)	1 (11)
Unspecified	28 (43)	20 (46)	5 (42)	3 (33)
**Patient characteristics**				
Average age, median [Q1,Q3]	62 [59, 69]	63 [59, 69]	62 [55, 65]	66 [63, 68]
Proportion male, median [Q1,Q3]	62 [53, 72]	63 [55, 70]	61 [51374]	58 [50, 60]
Severity of illness measure^a^				
APACHE II	23 (35)	13 (29.5)	7 (58)	3 (33)
APACHE III	2 (3)	1 (2)	0 (0)	1 (11)
APACHE IV	1 (2)	1 (2)	0 (0)	0 (0)
SAPS II	1 (2)	1 (2)	0 (0)	0 (0)
SAPS III	1 (2)	1 (2)	0 (0)	0 (0)
SOFA	6 (9)	4 (9)	1 (8)	1 (11)
Average APACHE II, median [Q1,Q3]	15 [11, 20]	15 [11, 18]	13 [11, 20]	20 [18, 21]
Average SOFA, median [Q1,Q3]	5 [4, 7]	4 [4, 5]	6 [6, 6]	9 [9, 9]

Values in the table are count (%) unless otherwise noted. The RCTs may be counted in several categories for each characteristic, so that the count (%) will not necessarily sum to 65 (100%)

Abbreviation: *ARF* Acute respiratory failure

^a^Multiple categories could be selected for each delirium RCT, so that the count (%) will not necessarily sum to 65 (100%)

^b^Europe includes Switzerland (1 prevention trial), Italy (1 prevention trial, 1 prevention/treatment trial), and Great Britain (2 prevention/treatment trials)

^c^Other includes Egypt (3), Netherlands (3), Turkey (3), South Korea (2), Thailand (2), Australia (1), Belgium (1), Chile (1), India (1), Saudia Arabia (1), and Serbia (1)

^d^Mixed ICU is a medical-surgical ICU

^e^Cardiac surgery and general surgery are mutually exclusive. If a trial recruited both cardiac and non-cardiac surgery patients, it was classified as general surgery. Surgery includes patients with a trauma designation

^f^Acute respiratory failure includes trials conducted among mechanically ventilated, acute respiratory, or acute respiratory distress syndrome patients

### Delirium assessments

The majority of RCTs (42, 65%) used the Confusion Assessment Method for the ICU (CAM-ICU) to assess delirium (Table 2). Assessments occurred once, twice, and more than twice per day in 23 (35%), 28 (43%), and 11 (17%) of eligible RCTs, respectively. The maximum duration for which delirium was assessed (i.e., the duration of follow-up) and was highly variable, with 3 days being the most common duration, used in 13 (20%) RCTs. Delirium was assessed for a maximum of 7 days in 31 (48%) RCTs, with 8 (12%) RCTs assessing delirium until ICU discharge. Delirium assessments were conducted only during the patients ICU stay for 41 (63%) RCTs, with a greater proportion of trials conducted among critically ill patients compared to surgery patients terminating delirium assessments at ICU discharge (14 of 17, 82% vs. 21 of 41, 51%, respectively). A single RCT reported a change in the frequency of delirium assessments following ICU discharge (twice daily during the ICU stay to daily while in the ward) [23].

**Table 2:** Characteristics of delirium assessments for the 65 delirium trials

	Overall	Prevention	Treatment	Both
	***n*** = 65	***n*** = 44	***n*** = 12	***n*** = 9
Delirium screening instrument^a^				
CAM-ICU	42 (65)	27 (61)	8 (67)	7 (78)
CAM	7 (11)	5 (11)	0 (0)	2 (22)
DSM Criteria	5 (8)	4 (9)	1 (8)	0 (0)
ICDSC	4 (6)	2 (5)	2 (17)	0 (0)
NEECHAM	4 (6)	3 (7)	1 (8)	0 (0)
Chart review	2 (3)	2 (5)	0 (0)	0 (0)
DSI	1 (2)	1 (2)	0 (0)	0 (0)
Frequency of assessments				
Daily	23 (35)	18 (41)	3 (25)	2 (22)
Twice daily	28 (43)	19 (43)	6 (50)	3 (33)
More than twice daily	11 (17)	5 (11)	2 (17)	4 (44)
Unclear	3 (5)	2 (5)	1 (8)	0 (0)
Maximum duration of delirium assessment				
3 days	13 (20)	11 (25)	2 (17)	0 (0)
46 days	9 (14)	6 (14)	1 (8)	2 (22)
7 days	9 (14)	7 (16)	1 (8)	1 (11)
814 days	6 (9)	2 (5)	4 (33)	0 (0)
2830 days	9 (14)	2 (5)	3 (25)	4 (44)
To ICU discharge	8 (12)	7 (16)	0 (0)	1 (11)
To hospital discharge	5 (8)	4 (9)	1 (8)	0 (0)
Other	2 (3)	2 (5)	0 (0)	0 (0)
Unclear	4 (6)	3 (7)	0 (0)	1 (11)
Delirium assessments terminated after ICU discharge^b^	41 (63)	29 (66)	6 (50)	6 (67)

Values in the table are count (%)

Abbreviations: *CAM* Confusion Assessment Method, *CAM-ICU* Confusion Assessment Method for the intensive care unit, *DSI* Delirium Symptom Interview, *ICDSC* Intensive Care Delirium Screening Checklist, *NEECHAM* Neelon-Champagne Confusion Scale, *DSM* Diagnostic and Statistical Manual

^a^The primary delirium assessment instrument reported

^b^Among the 24 trials that continued delirium assessments after ICU discharge, delirium was assessed using the Confusion Assessment Method (CAM) (21%, *n*=5), the CAM-ICU (42%, *n*=10), a combination of CAM and CAM-ICU (17%, *n*=4), NEECHAM (6%, *n*=2), DSM criteria (6%, *n*=2) or the Delirium Symptom Interview (3%, *n*=1)

### Risk of bias

Of the 65 RCTs, 14 (22%) had a high risk of bias for at least one of the 5 categories that were evaluated. High risk of bias for the 5 categories are presented in Additional File Figure 1 and Additional File Table 2, with highlights herein: 7 (11%) RCTs were categorized as high risk of bias due to lack of blinding and 5 (8%) for incomplete outcome data with respect to the primary delirium outcome.

### Delirium outcomes

Of the 65 RCTs, 61 (94%) reported a single primary delirium outcome and 4 reported delirium as a co-primary outcome. In addition, 29 (45%) RCTs reported a delirium-related outcome as a secondary outcome; with 20 and 8 reporting 1 or 2 secondary delirium outcomes, respectively. In the sections below, we report on the use of the four categories of delirium outcomes: delirium incidence, delirium composite outcome, delirium duration, and delirium severity, as well as the statistical methods applied to each outcome.

### Delirium incidence outcome

There were a total of 56 delirium incidence outcomes reported by 50 (77%) of the 65 trials; 42 (65%), 6 (9%), and 2 (3%) RCTs reported only a primary, both a primary and secondary, or only a secondary delirium incidence outcome, respectively. In 5 RCTs (8%), delirium incidence was evaluated at multiple time points (e.g., 14 and 28 days) within the same trial. We identified two definitions of delirium incidence, whether the patient ever met the criteria for delirium during follow-up and the presence/absence of delirium on each day during the follow-up.

The 56 primary or secondary delirium incidence outcomes were evaluated using 8 unique statistical methods (Table 3). The variation in statistical methods applied to delirium incidence outcomes was similar when comparing RCTs conducted among surgery vs. critically ill patients (6 unique statistical methods, respectively; Additional File Table 4). The most common statistical method, applied to 51 (91%) of 56 outcomes, defined a binary indicator for delirium and used a two-sample test for proportions (e.g., chi-square test or logistic regression) to compare delirium incidence across interventions.

**Table 3:** Statistical methods applied to delirium incidence

Statistical method	Overall^**a**^	Primary outcome^**a**^	Secondary outcome^**a**^
	***n*** = 56	***n*** = 48	***n*** = 8
Two-sample test for proportions^b^	51 (91)	45 (94)	6 (75)
Two-sample test for means^c^	1 (2)	1 (2)	0 (0)
Non-parametric test^d^	2 (4)	1 (2)	1 (13)
Binomial regression model^e^	1 (2)	0 (0)	1 (13)
Longitudinal regression model^f^	2 (4)	1 (2)	1 (13)
Survival analysis^g^	9 (16)	6 (13)	3 (38)
Competing risk survival analysis^h^	1 (2)	1 (2)	0 (0)
Joint model^i^	1 (2)	1 (2)	0 (0)

Values in the table are count (%). Several statistical methods may be reported for each outcome; therefore, column counts (%s) will not sum to the number of primary or secondary outcomes or 100%

^a^The sample size, *n*, reported as overall is the total number of delirium incidence outcomes, both primary and secondary, whereas the sample size reported for primary and secondary delirium incidence outcomes is the number of trials. A trial may report multiple delirium incidence outcomes, e.g., delirium incidence by 14 or 28 days as the primary and secondary outcomes, respectively. There were a total of 56 delirium incidence outcomes reported by 50 of the 65 trials; 42, 6, and 2 trials reported only a primary, both a primary and secondary, or only a secondary delirium incidence outcome, respectively

^b^Two-sample test for proportions includes two-sample test for proportions assuming normally distributed sample proportions, Fishers exact test, chi-square test, and logistic regression model

^c^Two-sample test for means includes two-sample *t* test, analysis of variance, or linear regression model

^d^Non-parametric test for continuous or ordinal outcomes includes Mann-Whitney test, Wilcoxon rank-sum test, Kruskal-Wallis test, and the proportional odds logistic regression model

^e^Binomial regression model defines the number of days with delirium as the binomial outcome and the number of days in the ICU as the offset/denominator

^f^Longitudinal regression model includes marginal longitudinal logistic regression models for daily delirium and random effects logistic regression models for daily delirium

^g^Survival analysis defined the outcome as time from randomization to delirium onset with patients censored at ICU discharge or death; statistical comparisons were made using the log-rank test or the Cox proportional hazards regression model

^h^Competing risk survival analysis defined the outcome as time from randomization to delirium onset with (i) patients censored at ICU discharge and death defined as a competing risk or (ii) ICU discharge and death defined as competing risks; statistical comparisons were made using the Fine and Gray competing risk model

^i^Joint model refers to the joint model for recurrent event outcomes (e.g., recurrent delirium events) with terminating event (e.g., ICU discharge or death) proposed by Rondeau [23]

As an alternative, the time to first positive delirium assessment was identified and the hazard of delirium was compared using standard and competing risk survival analysis for 10 (18%) outcomes. When using standard survival analysis methods, patients were censored at the end of the follow-up (e.g., 3 days), upon ICU discharge (for RCTs that did not assess delirium beyond discharge) or upon death. Death was considered a competing risk in the survival analysis for only 1 (10%) of the 10 outcomes [24].

The daily presence/absence of delirium during follow-up was compared across interventions in 4 RCTs (7%) via binomial regression (*n*=1) [25], longitudinal logistic regression models (*n*=2) [26, 27], or a joint model for recurrent days of delirium plus the terminating event of ICU discharge/death (*n*=1) [28, 29].

Only 4 (8%) of the 48 RCTs with a delirium incidence primary outcome reported conducting an analysis, primary or secondary, of delirium incidence that included adjustment for baseline variables [23, 27, 30, 31].

### Delirium composite outcome

Delirium-free days (DFD) and delirium- and coma-free days (DCFD) are composite outcomes, similar to ventilator-free days very commonly used in RCTs of mechanically ventilated patients in the ICU [32]. This composite outcome is often defined as the number of days that a patient is alive and free of delirium (or delirium and coma) during a fixed follow-up duration (e.g., 14 days), with patients who die during the follow-up often being assigned a value of 0 for this composite outcome. In trials where delirium is not assessed after ICU discharge, often the days between ICU discharge and the end of follow-up are assumed to be free of delirium. Delirium composite outcomes were reported in 11 (17%) RCTs, with 6 (9%), 2 (3%), and 3 (5%) RCTs reporting a delirium composite as only a primary only, both a primary and secondary (evaluated at different time points) and secondary only, respectively. A majority of the delirium composite outcomes were defined in delirium RCTs conducted on critically ill patients (8 of 11, 73%) (Additional File Table 5).

Five unique statistical methods were applied to the 13 delirium composite outcomes (Additional File Table 5). The most common method, applied to 11 (85%) of the 13 outcomes, was a non-parametric test to compare the distribution of the composite outcome across the intervention groups. The two-sample *t* test was used to compare the means of 4 (31%) composite outcomes, and Poisson regression was used for 2 (15%) composite outcomes (from the same RCT defined at days 8 and 30) [33]. The joint model, described above, was applied as a secondary analysis of the composite outcome in 1 (8%) RCT [34]. A single RCT was adjusted for baseline variables in the analysis of the delirium composite primary outcome [35].

### Delirium duration outcome

Delirium duration was a primary or secondary outcome for 6 (9%) and 18 (28%) of 65 RCTs, respectively (Additional File Table 3). The majority (14 of 18, 78%) of RCTs reporting delirium duration as a secondary outcome were prevention trials. Delirium duration was defined up to a fixed number of days (13, 54%), until ICU discharge (6, 25%), or until hospital discharge (4, 17%).

The 24 delirium duration outcomes were analyzed using 5 unique statistical methods (Additional File Table 6): a non-parametric test (12, 50%), two-sample test for means (9, 38%), survival analysis (3, 13%), Poisson regression (2, 8%) [36, 37], and two-sample test for proportions (1, 4%) [38]. The use of a non-parametric test or two-sample test for means occurred with similar frequency when comparing delirium RCTs conducted among surgery and critically ill patients (Additional File Table 6). A single RCT conducted an analysis of delirium duration that included adjustment for baseline variables [39].

### Delirium severity outcome

Delirium severity was the primary outcome for 8 (12%) RCTs (Additional File Table 3), with 5 of 12 (42%) treatment trials having delirium severity as the primary outcome. In addition, 8 (12%) RCTs had delirium severity as a secondary outcome, all of which were RCTs conducted among surgery patients and 7 (88%) of which were prevention trials (Additional File Table 7).

Delirium severity was compared across intervention groups using 2 approaches applying 3 unique statistical methods (Additional File Table 7). The first approach computed the worst delirium severity score during follow-up for each patient and applied a non-parametric test (10 of 16 outcomes, 63%) or a two-sample test for means (3, 19%). Alternatively, a longitudinal regression model was applied to the daily delirium severity scores (5, 31%). No RCT adjusted for baseline variables in the analysis of delirium severity.

### Mortality and ICU length of stay

Mortality and ICU length of stay (LOS) are common secondary outcomes, used in 20 (31%) and 31 (48%) of the 65 RCTs, respectively (Additional File Table 3). In addition, approximately 25% of delirium trials reported mortality and ICU LOS even when not named as primary or secondary outcomes. Compared to trials conducted among surgery patients, trials conducted among critically ill patients were more likely to include mortality (11 of 17, 65% vs. 8 of 41, 20%) and ICU LOS (11 of 17, 65% vs. 17 of 41, 41%) as secondary outcomes.

## Discussion

This systematic review focused on the design and analysis of delirium outcomes. We identified 65 RCTs conducted among ICU patients with a delirium-related primary outcome, the majority of which were delirium prevention trials, with considerable heterogeneity in both the maximum duration of participant follow-up and whether delirium assessments occurred after ICU discharge. To detect differences in delirium incidence across intervention groups, the most common delirium outcome, 8 unique statistical methods were used. Heterogeneity in statistical methods also occurred with less commonly used delirium outcomes, i.e., delirium composite, delirium duration, and delirium severity, with 5, 5, and 3 unique statistical methods reported, respectively. Heterogeneity in statistical methods was similar across the two main populations of patients enrolled in delirium RCTs; surgery and critically ill patients.

Heterogeneity across delirium RCTs is expected. Features of the target patient population and understanding of the proposed treatment mechanisms should drive choices for the design and selection of delirium outcome(s). Further, multiple statistical methods are often appropriate for analyzing the same delirium outcome and the choice of method(s) may depend on the accessibility of relevant statistical tools for both sample size estimation and data analysis. Given this issue and the goal of advancing methodologic recommendations, our findings support several key considerations for the design and analysis of delirium RCTs, as well as, highlight areas for future research including the need for developing statistical methods specific to the clinical features of delirium and obtaining consensus on the use of these methods among delirium clinical researchers (Table 4).

**Table 4:** Reporting and analysis of delirium outcomes in delirium prevention and treatment trials: problems identified and considerations for future research

Problem identified in the review	Considerations for future research
There is considerable heterogeneity in the design of delirium RCTs; including variation in the duration of follow-up, frequency of delirium assessments, whether delirium is assessed after ICU discharge and patient population being evaluated (e.g., cardiac surgery vs. critically ill patients)	Delirium outcome definitions should be explicitly defined: The definition should include the maximum duration of follow-up, the frequency of delirium assessments, whether delirium is assessed after ICU discharge, and how patient mortality is incorporated, or accounted for, in the outcome definition. The definition for delirium composite outcomes should include how mortality is incorporated and how delirium and coma status is defined after ICU discharge, if delirium assessments are terminated at ICU discharge. For example, a delirium RCT conducted among MV/ARF patients may define delirium incidence as whether a patient screens positive for delirium during at least one assessment while alive in the ICU within 14 days of randomization. The consensus among key stakeholders (patients, families, and clinicians) for primary and secondary delirium outcome definitions is warranted.
Delirium incidence and duration of delirium are most often compared across intervention groups using two-sample tests for proportions or means. When delirium assessments are terminated at ICU discharge with risk for post-discharge delirium or mortality rates are high, as expected in delirium RCTs conducted among critically ill ICU patients, comparisons of proportions or means may be misleading.	Statistical analysis methods for delirium outcomes should summarize censoring due to ICU discharge and the competing risk of death: Comparisons of ICU discharge or death across the interventions should be provided and alternative survival analysis methods should be considered, but have yet to be fully evaluated for delirium RCTs. Coma or deep sedation may be considered an additional censoring event; the impact of which has not been evaluated. Recurrent event survival methods may offer increased power to detect differences in delirium incidence across intervention groups in delirium prevention trials conducted among critically ill patients where delirium episodes may be recurring.
Delirium composite outcomes are common outcomes in RCTs targeting both prevention and treatment of delirium. In such RCTs, mortality may be ranked as the worst state and assigned a value of zero and if delirium assessments are terminated at ICU discharge, it may be assumed that patients are free of delirium after ICU discharge.	In general, the average of a rank-based delirium composite outcome is not directly interpretable: Non-parametric tests that focus on the ranking of the numerical values of the composite outcome measure should be used to make comparisons across intervention groups. Further evaluation of composite outcomes is warranted in delirium RCTs that terminate assessments at ICU discharge to understand the behavior of these outcomes (i.e., type I error rate), when interventions may impact both onset and duration of delirium, as well as the length of ICU stay and mortality.
Only 6 (9%) of 65 primary delirium outcomes were analyzed using methods that adjusted for baseline variables.	Adjusting for prognostic baseline variables for delirium may improve the precision of statistical comparisons of delirium outcomes across intervention groups (i.e., increase statistical power). Statistical approaches accounting for prognostic baseline variables have been developed for a wide range of outcome types (e.g., binary, time-to-event, and rank-based composites). The potential precision gains from these approaches have not been evaluated within delirium RCTs despite the availability of known risk factors commonly collected in delirium RCTs, including age, APACHE severity of illness score, and use of sedatives.

First, it is important to explicitly define delirium outcomes. Key features of a reported definition should include the maximum duration of follow-up, the frequency of delirium assessments, whether delirium is assessed after ICU discharge, and how patient mortality is incorporated, or accounted for, in the outcome. For example, a delirium RCT conducted among ARF patients may define delirium incidence as whether a patient screens positive for delirium at least once during any twice-daily assessment while alive in the ICU within 14 days of randomization. The definition of delirium composite outcomes should include how mortality is incorporated and how delirium and coma status is defined after ICU discharge, if delirium assessments are terminated at ICU discharge. Further, the consensus from key stakeholders (patients, families, and clinicians) on primary and secondary delirium outcome definitions is warranted and would improve the ability to harmonize results across delirium trials.

Second, statistical analysis methods for delirium outcomes should consider censoring due to ICU discharge and the competing risk of death. The occurrence of discharge creates a statistical and inferential issue known as informative censoring, as discharge can be correlated with delirium incidence, duration, and severity [40,43] and death precludes the occurrence of or resolution of delirium. The most frequently utilized two-sample test comparing the proportion of patients ever screened positive for delirium during follow-up (delirium incidence) or the mean duration of delirium may be sufficient to detect differences across intervention groups if delirium assessments occur after ICU discharge (or delirium is not expected after ICU discharge) and mortality rates are low, as expected in delirium RCTs conducted among surgery patients. However, when delirium assessments are terminated at ICU discharge with risk for post-discharge delirium or mortality rates are high, as expected in delirium RCTs conducted among critically ill ICU patients, comparisons of proportions or means may be misleading. For example, if patterns of mortality differ across intervention groups, a difference in the proportions may be detected even if the intervention had no impact on the incidence of delirium [12, 43]. Comparing the proportion of ever delirium patients defines the total effect, which includes the direct effect of the intervention on the incidence of delirium plus the effect mediated through death [41, 43, 44]. Comparisons of patterns of censoring or death across the interventions should be provided [45] and alternative survival analysis methods should be considered, but have yet to be fully evaluated for delirium RCTs [43]. Further, in delirium prevention trials conducted among critically ill patients, evaluating only the first occurrence of delirium ignores information from potentially recurring delirium episodes [28]. For this reason, the use of recurrent event survival methods offers an appealing alternative approach and is currently being extended to include the ability to account separately for ICU discharge and death as censoring events [16, 29, 46]. One additional type of censoring that should be considered in delirium RCTs conducted among critically ill patients is coma or deep sedation. Patients may be considered not at risk for delirium, with no delirium assessment conducted, during periods of coma or deep sedation [28], or coma or deep sedation may be considered part of the continuum of the delirium experience and included in the delirium outcome definition [47]. To our knowledge, the impact of treating coma or deep sedation as a censoring event has not been evaluated.

Third, delirium composite outcomes (e.g., days free of delirium and coma to 28 days) are common outcomes in RCTs targeting both prevention and treatment of delirium in critically ill patients. In such RCTs, mortality may be ranked as the worst state and assigned a value of zero. In such cases, the average delirium composite outcome is not directly interpretable, requiring additional reporting of the components of the composite outcome (e.g., as secondary outcomes) [48]. Moreover, comparisons across intervention groups should be made using non-parametric tests that focus on the ranking of the numerical values of the composite outcome measure [48]. Further evaluation of composite outcomes is warranted in delirium RCTs that terminate assessments at ICU discharge to understand the behavior of these outcomes (i.e., type I error rate), when interventions may impact both onset and duration of delirium, as well as the length of ICU stay and mortality.

Lastly, baseline variables, which are prognostic for delirium incidence (e.g., age, APACHE II severity of illness score, receiving a sedative drug [49]), are often collected in delirium RCTs. However, only 6 (9%) of the 65 RCTs conducted an analysis, primary or secondary, of the primary delirium outcome that adjusted for baseline variables. Adjusting for baseline variables may improve the precision of statistical comparisons of delirium outcomes across intervention groups (i.e., increase statistical power) [50]. Robust statistical methods for baseline variable adjustment have been developed for a wide range of outcome types (e.g., binary, time to event, and rank-based composites) [51,55]. Exploring the utility of baseline variable adjustment in delirium RCTs is warranted.

Our systematic review has potential limitations. First, there may be errors or uncertainty (due to ambiguity in reporting) in the data abstraction. We sought to minimize by duplicate and independent data abstraction and carefully resolving any discrepancies, as well as utilizing data extractors with training in epidemiology and biostatistics. Second, it is possible that our systematic search or screening process inadvertently omitted some eligible RCTs. However, such omissions are unlikely to have been systematic, and given the size of our review and recurrent observations, is not expected to meaningfully alter our conclusions. Third, we chose to exclude trial registries from the systematic review. We did this so we could capture the primary and secondary analyses actually conducted (rather than those planned/proposed) for each delirium outcome since there are known instances of deviations (sometimes not reported clearly) of actual report vs. trial registry. However, we did review any Appendices/Supplementary Material (including study protocol) when included with the published trial when data elements of interest were not presented in the main manuscript. Fourth, our data collection for statistical methods did not include screening for adherence to CONSORT recommendations on reporting within RCTs [56]. For instance, analyses for binary outcomes should provide both a treatment effect and associated confidence interval with CONSORT recommending reporting both absolute risk difference and relative risk estimates. Our categories of statistical methods for two-sample tests for proportions include approaches, e.g., Fishers exact test or chi-square test, that do not necessarily adhere to these recommendations. Lastly, no formal consensus process or methodology was used to create our list of considerations with targets for future work since the focus of the paper is reporting the findings of the systematic review.

## Conclusions

Specification of delirium outcome definitions and statistical analysis methods to compare intervention groups require careful consideration of the duration of follow-up, ability to assess delirium after ICU discharge, and expectation of patient mortality. Creating uniform standards for statistical analyses and reporting in delirium RCTs will improve the quality of individual trials and the ability to harmonize results across trials. Further evaluation and development of statistical methods are warranted to promote the selection of appropriate statistical analysis methods.

## Supplementary Information

**Additional file 1: Section 1.** PRISMA checklist. **Section 2.** Search strategy. **Section 3.** Inclusion criteria. **Section 4.** Delirium outcome categories. **Section 5.** Statistical methods categories. **Table 1.** Individual study characteristics of the 65 delirium trials. **Table 2.** Individual study risk of bias assessments. **Table 3.** Frequency of primary and secondary delirium outcomes, mortality and ICU length of stay in the 65 delirium trials. **Table 4.** Statistical methods applied to delirium incidence, separately for delirium RCTs conducted among critically ill and surgery patients. **Table 5.** Statistical methods applied to the delirium composite, for all trials with a delirium composite outcome and separately for trials conducted among critically ill and surgery patients. **Table 6.** Statistical methods applied to delirium duration, for all trials with a delirium duration outcome and separately for trials conducted among critically ill and surgery patients. **Table 7.** Statistical methods applied to delirium severity, for all trials with a delirium severity outcome and separately for trials conducted among critically ill and surgery patients. **Figure 1.** Risk of bias analysis.

## Data Availability

The datasets generated and/or analyzed during the current study are not publicly available but are available from the corresponding author on reasonable request.

## Abbreviations

ICU: Intensive care unit
RCT: Randomized controlled trial
NIDUS: Network for Investigation of Delirium: Unifying Scientists
MV: Mechanically ventilated
ARF: Acute respiratory failure
ARDS: Acute respiratory distress syndrome
DFD: Delirium-free days
DCFD: Delirium- and coma-free days
LOS: Length of stay
