# Supplementary material for: Heterogeneity in design and analysis of ICU delirium randomized trials: a systematic review
Source: Trials. 2021 May 20;22:354. doi: 10.1186/s13063-021-05299-1 (PMC8136095; doi:10.1186/s13063-021-05299-1)
Supplement: Supplementary file 1 — Additional file 1: Section 1. PRISMA checklist. Section 2. Search strategy. Section 3. Inclusion criteria. Section 4. Delirium outcome categories. Section 5. Statistical methods categories. Table 1. Individual study characteristics of the 65 delirium trials. Table 2. Individual study risk of bias assessments. Table 3. Frequency of primary and secondary delirium outcomes, mortality and ICU length of stay in the 65 delirium trials. Table 4. Statistical methods applied to delirium incidence, separately for delirium RCTs conducted among critically ill and surgery patients. Table 5. Statistical methods applied to the delirium composite, for all trials with a delirium composite outcome and separately for trials conducted among critically ill and surgery patients. Table 6. Statistical methods applied to delirium duration, for all trials with a delirium duration outcome and separately for trials conducted among critically ill and surgery patients. Table 7. Statistical methods applied to delirium severity, for all trials with a delirium severity outcome and separately for trials conducted among critically ill and surgery patients. Figure 1. Risk of bias analysis. [file 13063_2021_5299_MOESM1_ESM.docx]

**Heterogeneity in design and analysis of ICU delirium randomized trials:**

**a systematic review**

Elizabeth Colantuoni, Mounica Koneru, Narjes Akhlaghi, Ximin Li, Mohamed O. Hashem, Victor D Dinglas, Karin J. Neufeld, Michael O. Harhay, Dale M. Needham

**Additional File**

**Additional File Section 1: PRISMA checklist**

| **Section/topic** | | **#** | | **Checklist item** | | **Reported on page #** | |
| --- | --- | --- | --- | --- | --- | --- | --- |
| **TITLE** | | | | | |  | |
| Title | | 1 | | Identify the report as a systematic review, meta-analysis, or both. | | 1 | |
| **ABSTRACT** | | | | | |  | |
| Structured summary | | 2 | | Provide a structured summary including, as applicable: background; objectives; data sources; study eligibility criteria, participants, and interventions; study appraisal and synthesis methods; results; limitations; conclusions and implications of key findings; systematic review registration number. | | 2-3 | |
| **INTRODUCTION** | | | | | |  | |
| Rationale | | 3 | | Describe the rationale for the review in the context of what is already known. | | 5 | |
| Objectives | | 4 | | Provide an explicit statement of questions being addressed with reference to participants, interventions, comparisons, outcomes, and study design (PICOS). | | 5 | |
| **METHODS** | | | | | |  | |
| Protocol and registration | | 5 | | Indicate if a review protocol exists, if and where it can be accessed (e.g., Web address), and, if available, provide registration information including registration number. | | 6 | |
| Eligibility criteria | | 6 | | Specify study characteristics (e.g., PICOS, length of follow-up) and report characteristics (e.g., years considered, language, publication status) used as criteria for eligibility, giving rationale. | | 6 | |
| Information sources | | 7 | | Describe all information sources (e.g., databases with dates of coverage, contact with study authors to identify additional studies) in the search and date last searched. | | 6 | |
| Search | | 8 | | Present full electronic search strategy for at least one database, including any limits used, such that it could be repeated. | | Additional File Section 2 | |
| Study selection | | 9 | | State the process for selecting studies (i.e., screening, eligibility, included in systematic review, and, if applicable, included in the meta-analysis). | | 6, Additional File Section 3 | |
| Data collection process | | 10 | | Describe method of data extraction from reports (e.g., piloted forms, independently, in duplicate) and any processes for obtaining and confirming data from investigators. | | 7 | |
| Data items | | 11 | | List and define all variables for which data were sought (e.g., PICOS, funding sources) and any assumptions and simplifications made. | | 7, Additional File Sections 4 and 5 | |
| Risk of bias in individual studies | | 12 | | Describe methods used for assessing risk of bias of individual studies (including specification of whether this was done at the study or outcome level), and how this information is to be used in any data synthesis. | | 7 | |
| Summary measures | | 13 | | State the principal summary measures (e.g., risk ratio, difference in means). | | 7-8 | |
| Synthesis of results | | 14 | | Describe the methods of handling data and combining results of studies, if done, including measures of consistency (e.g., I^2^) for each meta-analysis. | | 7-8 | |
| Section/topic | | # | | Checklist item | | Reported on page # | |
| Risk of bias across studies | | 15 | | Specify any assessment of risk of bias that may affect the cumulative evidence (e.g., publication bias, selective reporting within studies). | | NA | |
| Additional analyses | | 16 | | Describe methods of additional analyses (e.g., sensitivity or subgroup analyses, meta-regression), if done, indicating which were pre-specified. | | 7-8 | |
| **RESULTS** | | | | | |  | |
| Study selection | | 17 | | Give numbers of studies screened, assessed for eligibility, and included in the review, with reasons for exclusions at each stage, ideally with a flow diagram. | | 8 | |
| Study characteristics | | 18 | | For each study, present characteristics for which data were extracted (e.g., study size, PICOS, follow-up period) and provide the citations. | | Additional File Table 1 | |
| Risk of bias within studies | | 19 | | Present data on risk of bias of each study and, if available, any outcome level assessment (see item 12). | | Additional File Table 2 and Figure 2 | |
| Results of individual studies | | 20 | | For all outcomes considered (benefits or harms), present, for each study: (a) simple summary data for each intervention group (b) effect estimates and confidence intervals, ideally with a forest plot. | | NA | |
| Synthesis of results | | 21 | | Present results of each meta-analysis done, including confidence intervals and measures of consistency. | | 8-13 | |
| Risk of bias across studies | | 22 | | Present results of any assessment of risk of bias across studies (see Item 15). | | NA | |
| Additional analysis | | 23 | | Give results of additional analyses, if done (e.g., sensitivity or subgroup analyses, meta-regression [see Item 16]). | | NA | |
| **DISCUSSION** | | | | | |  | |
| Summary of evidence | | 24 | | Summarize the main findings including the strength of evidence for each main outcome; consider their relevance to key groups (e.g., healthcare providers, users, and policy makers). | | 13 | |
| Limitations | | 25 | | Discuss limitations at study and outcome level (e.g., risk of bias), and at review-level (e.g., incomplete retrieval of identified research, reporting bias). | | 15 | |
| Conclusions | | 26 | | Provide a general interpretation of the results in the context of other evidence, and implications for future research. | | 13-15 | |
| **FUNDING** | | | | | |  | |
| Funding | | 27 | | Describe sources of funding for the systematic review and other support (e.g., supply of data); role of funders for the systematic review. | | 17 | |

**Additional File Section 2: Search strategy**

| **Aim** | A rigorous systematic review will be conducted to identify endpoints used, their definitions, and statistical analysis methods in delirium prevention  treatment RCTs in critically ill patients (adults). |
| --- | --- |
| **Concepts** | Aims document: critical illness, delirium and RCT |
| **Databases** | PubMed, The Cochrane Library, Embase, CINAHL Plus, PsycINFO, Scopus, Web of Science, ClinicalTrials.gov |
| **Trial Registries** | ClinicalTrials.gov, WHO Trials Registry |
| **Limits** | Humans |
| **Time Frame** | TBD |
| **Reference Manager** | Reference Manager |
| **Date Run** | 7/8/2019 |
| **Prepared by** | Carrie Price |
| **PRESS Reviewed by** | Blair Anton |
| **Total Number of Results** | 15,242 |
| **Duplicates Removed** | 6,389 |
| **Remaining Results** | 8,853 |

# PubMed: critical illness, delirium, RCTs*

*Cochrane Highly Sensitive Search Strategy for identifying randomized trials in MEDLINE: sensitivity maximizing version (<http://handbook-5-1.cochrane.org/chapter_6/box_6_4_a_cochrane_hsss_2008_sensmax_pubmed.htm>) with additions

("acute lung injury"[mesh] OR "burn units"[mesh] OR "critical care"[mesh:noexp] OR "critical illness"[mesh] OR "intensive care units"[mesh:noexp] OR "intubation"[mesh:noexp] OR "intubation, intratracheal"[mesh:noexp] OR "multiple organ failure"[mesh] OR "respiratory care units"[mesh] OR "respiration, artificial"[mesh:noexp] OR "respiratory distress syndrome, adult"[mesh] OR "sepsis"[mesh:noexp] OR "shock"[mesh:noexp] OR "shock, septic"[mesh] OR "ventilators, mechanical"[mesh] OR "bacteremia"[mesh] OR "endotoxemia"[mesh] OR "hemorrhagic septicemia"[mesh] OR "fungemia"[mesh] OR "candidemia"[mesh] OR "parasitemia"[mesh] OR "viremia"[mesh] OR "acute lung injuries"[tw] OR "acute lung injury"[tw] OR "ards"[tw] OR "artificial respiration"[tw] OR "artificial ventilation"[tw] OR "bacteremia"[tw] OR "bacteremia"[tw] OR "bacteremias"[tw] OR "bacteremic"[tw] OR "bacteremics"[tw] OR "blood poisoning"[tw] OR "blood poisonings"[tw] OR "burn unit"[tw] OR "burn units"[tw] OR "candidemia"[tw] OR "critical care"[tw] OR "critical illness"[tw] OR "critical illnesses"[tw] OR "critically ill"[tw] OR "endotoxemia"[tw] OR "endotoxemia"[tw] OR "endotoxemias"[tw] OR "endotoxemic"[tw] OR "endotoxemics"[tw] OR "fungemia"[tw] OR "i c u "[tw] OR "icu"[tw] OR "intensive care"[tw] OR "multi organ dysfunction"[tw] OR "multi organ dysfunctions"[tw] OR "multi organ failure"[tw] OR "multi organ failures"[tw] OR "multi system organ dysfunction"[tw] OR "multi system organ dysfunctions"[tw] OR "multi system organ failure"[tw] OR "multi system organ failures"[tw] OR "multiorgan dysfunction"[tw] OR "multiorgan dysfunctions"[tw] OR "multiorgan failure"[tw] OR "multiorgan failures"[tw] OR "multiple organ dysfunction"[tw] OR "multiple organ dysfunctions"[tw] OR "multiple organ failure"[tw] OR "multiple organ failures"[tw] OR "multisystem organ dysfunction"[tw] OR "multisystem organ dysfunctions"[tw] OR "multisystem organ failure"[tw] OR "multisystem organ failures"[tw] OR "pyaemia"[tw] OR "pyaemias"[tw] OR "pyaemic"[tw] OR "pyaemics"[tw] OR "pyemia"[tw] OR "pyemias"[tw] OR "pyemic"[tw] OR "pyemics"[tw] OR "pyohaemia"[tw] OR "pyohaemias"[tw] OR "pyohaemic"[tw] OR "pyohemia"[tw] OR "pyohemias"[tw] OR "pyohemic"[tw] OR "pyohemics"[tw] OR "pyohemics"[tw] OR "respiratory care unit"[tw] OR "respiratory care units"[tw] OR "respiratory distress syndrome"[tw] OR "respiratory distress syndromes"[tw] OR "sepsis"[tw] OR "septic"[tw] OR "septicaemia"[tw] OR "septicaemias"[tw] OR "septicaemic"[tw] OR "septicaemics"[tw] OR "septicemia"[tw] OR "septicemias"[tw] OR "shock"[tw] OR "viremia"[tw] OR "viremias"[tw] OR "viremic"[tw] OR "viremics"[tw] OR bacillaemi*[tw] OR bacillemi*[tw] OR bacteraemi*[tw] OR candidaemi*[tw] OR candidemi*[tw] OR endotoxaemi*[tw] OR fungaemi*[tw] OR fungemi*[tw] OR intubat*[tw] OR mechanical ventilat*[tw] OR mechanically ventilat*[tw] OR parasitaemi*[tw] OR parasitemi*[tw])

AND

("confusion"[mesh] OR "delirium"[mesh] OR "emergence delirium"[mesh] OR "agitated emergence"[tw] OR "altered consciousness"[tw] OR "altered mental state"[tw] OR "altered mental states"[tw] OR "altered mental status"[tw] OR "delire"[tw] OR "delirious"[tw] OR "delirious"[tw] OR "dis orientation"[tw] OR "dis orientations"[tw] OR "dis oriented"[tw] OR "emergence agitation"[tw] OR "emergence agitations"[tw] OR "emergence excitement"[tw] OR "emergence excitements"[tw] OR "mental status change"[tw] OR "mental status changes"[tw] OR "organic brain syndrome"[tw] OR "organic brain syndromes"[tw] OR "post anaesthetic excitement"[tw] OR "post anaesthetic excitements"[tw] OR "post anesthetic excitement"[tw] OR "post anesthetic excitements"[tw] OR "post anaesthesia excitement"[tw] OR "post anaesthesia excitements"[tw] OR "post anesthesia excitement"[tw] OR "post anesthesia excitements"[tw] OR "post operative agitation"[tw] OR "post operative excitement"[tw] OR "post surgical agitation"[tw] OR "post surgical excitement"[tw] OR "postanaesthetic excitement"[tw] OR "postanaesthetic excitements"[tw] OR "postanesthetic excitement"[tw] OR "postanesthetic excitements"[tw] OR "postanaesthesia excitement"[tw] OR "postanaesthesia excitements"[tw] OR "postanesthesia excitement"[tw] OR "postanesthesia excitements"[tw] OR "postoperative agitation"[tw] OR "postoperative excitement"[tw] OR "postsurgical agitation"[tw] OR "postsurgical excitement"[tw] OR confusion*[tw] OR delerium*[tw] OR deliria*[tw] OR delirium*[tw] OR disorient*[tw] OR hallucinat*[tw] OR illusion*[tw] OR illusor*[tw])

AND

("randomized controlled trial"[pt] OR "controlled clinical trial"[pt] OR "randomized"[tiab] OR "randomised"[tiab] OR "random"[tiab] OR "placebo"[tiab] OR "drug therapy"[sh] OR "randomly"[tiab] OR "trial"[tiab] OR "groups"[tiab]) NOT ("animals"[mesh] NOT ("animals"[mesh] AND "humans"[mesh]))

1,795 results

# The Cochrane Library

([mh "acute lung injury"] OR [mh "burn units"] OR [mh ^"critical care"] OR [mh "critical illness"] OR [mh ^"intensive care units"] OR [mh ^"intubation"] OR [mh ^"intubation, intratracheal"] OR [mh "multiple organ failure"] OR [mh "respiratory care units"] OR [mh ^"respiration, artificial"] OR [mh "respiratory distress syndrome, adult"] OR [mh ^"sepsis"] OR [mh ^"shock"] OR [mh "shock, septic"] OR [mh "ventilators, mechanical"] OR [mh "bacteremia"] OR [mh "endotoxemia"] OR [mh "hemorrhagic septicemia"] OR [mh "fungemia"] OR [mh "candidemia"] OR [mh "parasitemia"] OR [mh "viremia"] OR "acute lung injuries":ti,ab,kw OR "acute lung injury":ti,ab,kw OR "ards":ti,ab,kw OR "artificial respiration":ti,ab,kw OR "artificial ventilation":ti,ab,kw OR "bacteremia":ti,ab,kw OR "bacteremia":ti,ab,kw OR "bacteremias":ti,ab,kw OR "bacteremic":ti,ab,kw OR "bacteremics":ti,ab,kw OR "blood poisoning":ti,ab,kw OR "blood poisonings":ti,ab,kw OR "burn unit":ti,ab,kw OR "burn units":ti,ab,kw OR "candidemia":ti,ab,kw OR "critical care":ti,ab,kw OR "critical illness":ti,ab,kw OR "critical illnesses":ti,ab,kw OR "critically ill":ti,ab,kw OR "endotoxemia":ti,ab,kw OR "endotoxemia":ti,ab,kw OR "endotoxemias":ti,ab,kw OR "endotoxemic":ti,ab,kw OR "endotoxemics":ti,ab,kw OR "fungemia":ti,ab,kw OR "i c u ":ti,ab,kw OR "icu":ti,ab,kw OR "intensive care":ti,ab,kw OR "multi organ dysfunction":ti,ab,kw OR "multi organ dysfunctions":ti,ab,kw OR "multi organ failure":ti,ab,kw OR "multi organ failures":ti,ab,kw OR "multi system organ dysfunction":ti,ab,kw OR "multi system organ dysfunctions":ti,ab,kw OR "multi system organ failure":ti,ab,kw OR "multi system organ failures":ti,ab,kw OR "multiorgan dysfunction":ti,ab,kw OR "multiorgan dysfunctions":ti,ab,kw OR "multiorgan failure":ti,ab,kw OR "multiorgan failures":ti,ab,kw OR "multiple organ dysfunction":ti,ab,kw OR "multiple organ dysfunctions":ti,ab,kw OR "multiple organ failure":ti,ab,kw OR "multiple organ failures":ti,ab,kw OR "multisystem organ dysfunction":ti,ab,kw OR "multisystem organ dysfunctions":ti,ab,kw OR "multisystem organ failure":ti,ab,kw OR "multisystem organ failures":ti,ab,kw OR "pyaemia":ti,ab,kw OR "pyaemias":ti,ab,kw OR "pyaemic":ti,ab,kw OR "pyaemics":ti,ab,kw OR "pyemia":ti,ab,kw OR "pyemias":ti,ab,kw OR "pyemic":ti,ab,kw OR "pyemics":ti,ab,kw OR "pyohaemia":ti,ab,kw OR "pyohaemias":ti,ab,kw OR "pyohaemic":ti,ab,kw OR "pyohemia":ti,ab,kw OR "pyohemias":ti,ab,kw OR "pyohemic":ti,ab,kw OR "pyohemics":ti,ab,kw OR "pyohemics":ti,ab,kw OR "respiratory care unit":ti,ab,kw OR "respiratory care units":ti,ab,kw OR "respiratory distress syndrome":ti,ab,kw OR "respiratory distress syndromes":ti,ab,kw OR "sepsis":ti,ab,kw OR "septic":ti,ab,kw OR "septicaemia":ti,ab,kw OR "septicaemias":ti,ab,kw OR "septicaemic":ti,ab,kw OR "septicaemics":ti,ab,kw OR "septicemia":ti,ab,kw OR "septicemias":ti,ab,kw OR "shock":ti,ab,kw OR "viremia":ti,ab,kw OR "viremias":ti,ab,kw OR "viremic":ti,ab,kw OR "viremics":ti,ab,kw OR bacillaemi*:ti,ab,kw OR bacillemi*:ti,ab,kw OR bacteraemi*:ti,ab,kw OR candidaemi*:ti,ab,kw OR candidemi*:ti,ab,kw OR endotoxaemi*:ti,ab,kw OR fungaemi*:ti,ab,kw OR fungemi*:ti,ab,kw OR intubat*:ti,ab,kw OR "mechanical ventilat*":ti,ab,kw OR "mechanically ventilat*":ti,ab,kw OR parasitaemi*:ti,ab,kw OR parasitemi*:ti,ab,kw)

AND

([mh "confusion"] OR [mh "delirium"] OR [mh "emergence delirium"] OR "agitated emergence":ti,ab,kw OR "altered consciousness":ti,ab,kw OR "altered mental state":ti,ab,kw OR "altered mental states":ti,ab,kw OR "altered mental status":ti,ab,kw OR "delire":ti,ab,kw OR "delirious":ti,ab,kw OR "delirious":ti,ab,kw OR "dis orientation":ti,ab,kw OR "dis orientations":ti,ab,kw OR "dis oriented":ti,ab,kw OR "emergence agitation":ti,ab,kw OR "emergence agitations":ti,ab,kw OR "emergence excitement":ti,ab,kw OR "emergence excitements":ti,ab,kw OR "mental status change":ti,ab,kw OR "mental status changes":ti,ab,kw OR "organic brain syndrome":ti,ab,kw OR "organic brain syndromes":ti,ab,kw OR "post anaesthetic excitement":ti,ab,kw OR "post anaesthetic excitements":ti,ab,kw OR "post anesthetic excitement":ti,ab,kw OR "post anesthetic excitements":ti,ab,kw OR "post anaesthesia excitement":ti,ab,kw OR "post anaesthesia excitements":ti,ab,kw OR "post anesthesia excitement":ti,ab,kw OR "post anesthesia excitements":ti,ab,kw OR "post operative agitation":ti,ab,kw OR "post operative excitement":ti,ab,kw OR "post surgical agitation":ti,ab,kw OR "post surgical excitement":ti,ab,kw OR "postanaesthetic excitement":ti,ab,kw OR "postanaesthetic excitements":ti,ab,kw OR "postanesthetic excitement":ti,ab,kw OR "postanesthetic excitements":ti,ab,kw OR "postanaesthesia excitement":ti,ab,kw OR "postanaesthesia excitements":ti,ab,kw OR "postanesthesia excitement":ti,ab,kw OR "postanesthesia excitements":ti,ab,kw OR "postoperative agitation":ti,ab,kw OR "postoperative excitement":ti,ab,kw OR "postsurgical agitation":ti,ab,kw OR "postsurgical excitement":ti,ab,kw OR confusion*:ti,ab,kw OR delerium*:ti,ab,kw OR deliria*:ti,ab,kw OR delirium*:ti,ab,kw OR disorient*:ti,ab,kw OR hallucinat*:ti,ab,kw OR illusion*:ti,ab,kw OR illusor*:ti,ab,kw)

1,285 trials

# Embase

('acute lung injury'/de OR 'burn unit'/de OR 'critical illness'/de OR 'intensive care'/de OR 'intubation'/de OR 'endotracheal intubation'/de OR 'multiple organ failure'/de OR 'artificial ventilation'/de OR 'adult respiratory distress syndrome'/de OR 'sepsis'/de OR 'shock'/de OR 'septic shock'/de OR 'mechanical ventilator'/de OR 'bacteremia'/de OR 'endotoxemia'/de OR 'hemorrhagic septicemia'/de OR 'fungemia'/de OR 'candidemia'/de OR 'parasitemia'/de OR 'viremia'/de OR 'acute lung injuries':ti,ab,kw OR 'acute lung injury':ti,ab,kw OR 'ards':ti,ab,kw OR 'artificial respiration':ti,ab,kw OR 'artificial ventilation':ti,ab,kw OR 'bacteremia':ti,ab,kw OR 'bacteremia':ti,ab,kw OR 'bacteremias':ti,ab,kw OR 'bacteremic':ti,ab,kw OR 'bacteremics':ti,ab,kw OR 'blood poisoning':ti,ab,kw OR 'blood poisonings':ti,ab,kw OR 'burn unit':ti,ab,kw OR 'burn units':ti,ab,kw OR 'candidemia':ti,ab,kw OR 'critical care':ti,ab,kw OR 'critical illness':ti,ab,kw OR 'critical illnesses':ti,ab,kw OR 'critically ill':ti,ab,kw OR 'endotoxemia':ti,ab,kw OR 'endotoxemia':ti,ab,kw OR 'endotoxemias':ti,ab,kw OR 'endotoxemic':ti,ab,kw OR 'endotoxemics':ti,ab,kw OR 'fungemia':ti,ab,kw OR 'i c u ':ti,ab,kw OR 'icu':ti,ab,kw OR 'intensive care':ti,ab,kw OR 'multi organ dysfunction':ti,ab,kw OR 'multi organ dysfunctions':ti,ab,kw OR 'multi organ failure':ti,ab,kw OR 'multi organ failures':ti,ab,kw OR 'multi system organ dysfunction':ti,ab,kw OR 'multi system organ dysfunctions':ti,ab,kw OR 'multi system organ failure':ti,ab,kw OR 'multi system organ failures':ti,ab,kw OR 'multiorgan dysfunction':ti,ab,kw OR 'multiorgan dysfunctions':ti,ab,kw OR 'multiorgan failure':ti,ab,kw OR 'multiorgan failures':ti,ab,kw OR 'multiple organ dysfunction':ti,ab,kw OR 'multiple organ dysfunctions':ti,ab,kw OR 'multiple organ failure':ti,ab,kw OR 'multiple organ failures':ti,ab,kw OR 'multisystem organ dysfunction':ti,ab,kw OR 'multisystem organ dysfunctions':ti,ab,kw OR 'multisystem organ failure':ti,ab,kw OR 'multisystem organ failures':ti,ab,kw OR 'pyaemia':ti,ab,kw OR 'pyaemias':ti,ab,kw OR 'pyaemic':ti,ab,kw OR 'pyaemics':ti,ab,kw OR 'pyemia':ti,ab,kw OR 'pyemias':ti,ab,kw OR 'pyemic':ti,ab,kw OR 'pyemics':ti,ab,kw OR 'pyohaemia':ti,ab,kw OR 'pyohaemias':ti,ab,kw OR 'pyohaemic':ti,ab,kw OR 'pyohemia':ti,ab,kw OR 'pyohemias':ti,ab,kw OR 'pyohemic':ti,ab,kw OR 'pyohemics':ti,ab,kw OR 'pyohemics':ti,ab,kw OR 'respiratory care unit':ti,ab,kw OR 'respiratory care units':ti,ab,kw OR 'respiratory distress syndrome':ti,ab,kw OR 'respiratory distress syndromes':ti,ab,kw OR 'sepsis':ti,ab,kw OR 'septic':ti,ab,kw OR 'septicaemia':ti,ab,kw OR 'septicaemias':ti,ab,kw OR 'septicaemic':ti,ab,kw OR 'septicaemics':ti,ab,kw OR 'septicemia':ti,ab,kw OR 'septicemias':ti,ab,kw OR 'shock':ti,ab,kw OR 'viremia':ti,ab,kw OR 'viremias':ti,ab,kw OR 'viremic':ti,ab,kw OR 'viremics':ti,ab,kw OR bacillaemi*:ti,ab,kw OR bacillemi*:ti,ab,kw OR bacteraemi*:ti,ab,kw OR candidaemi*:ti,ab,kw OR candidemi*:ti,ab,kw OR endotoxaemi*:ti,ab,kw OR fungaemi*:ti,ab,kw OR fungemi*:ti,ab,kw OR intubat*:ti,ab,kw OR 'mechanical ventilat*':ti,ab,kw OR 'mechanically ventilat*':ti,ab,kw OR parasitaemi*:ti,ab,kw OR parasitemi*:ti,ab,kw)

AND

('confusion'/de OR 'delirium'/de OR 'agitated emergence':ti,ab,kw OR 'altered consciousness':ti,ab,kw OR 'altered mental state':ti,ab,kw OR 'altered mental states':ti,ab,kw OR 'altered mental status':ti,ab,kw OR 'delire':ti,ab,kw OR 'delirious':ti,ab,kw OR 'delirious':ti,ab,kw OR 'dis orientation':ti,ab,kw OR 'dis orientations':ti,ab,kw OR 'dis oriented':ti,ab,kw OR 'emergence agitation':ti,ab,kw OR 'emergence agitations':ti,ab,kw OR 'emergence excitement':ti,ab,kw OR 'emergence excitements':ti,ab,kw OR 'mental status change':ti,ab,kw OR 'mental status changes':ti,ab,kw OR 'organic brain syndrome':ti,ab,kw OR 'organic brain syndromes':ti,ab,kw OR 'post anaesthetic excitement':ti,ab,kw OR 'post anaesthetic excitements':ti,ab,kw OR 'post anesthetic excitement':ti,ab,kw OR 'post anesthetic excitements':ti,ab,kw OR 'post anaesthesia excitement':ti,ab,kw OR 'post anaesthesia excitements':ti,ab,kw OR 'post anesthesia excitement':ti,ab,kw OR 'post anesthesia excitements':ti,ab,kw OR 'post operative agitation':ti,ab,kw OR 'post operative excitement':ti,ab,kw OR 'post surgical agitation':ti,ab,kw OR 'post surgical excitement':ti,ab,kw OR 'postanaesthetic excitement':ti,ab,kw OR 'postanaesthetic excitements':ti,ab,kw OR 'postanesthetic excitement':ti,ab,kw OR 'postanesthetic excitements':ti,ab,kw OR 'postanaesthesia excitement':ti,ab,kw OR 'postanaesthesia excitements':ti,ab,kw OR 'postanesthesia excitement':ti,ab,kw OR 'postanesthesia excitements':ti,ab,kw OR 'postoperative agitation':ti,ab,kw OR 'postoperative excitement':ti,ab,kw OR 'postsurgical agitation':ti,ab,kw OR 'postsurgical excitement':ti,ab,kw OR confusion*:ti,ab,kw OR delerium*:ti,ab,kw OR deliria*:ti,ab,kw OR delirium*:ti,ab,kw OR disorient*:ti,ab,kw OR hallucinat*:ti,ab,kw OR illusion*:ti,ab,kw OR illusor*:ti,ab,kw)

AND

('randomized controlled trial'/de OR 'controlled clinical trial'/de OR 'randomized':ti,ab OR 'randomised':ti,ab OR 'random':ti,ab OR 'placebo':ti,ab OR 'drug therapy'/de OR 'randomly':ti,ab OR 'trial':ti,ab OR 'groups':ti,ab) NOT ('animal'/exp NOT ('animal'/exp AND 'human'/exp))

3,698 results

# CINAHL Plus

(MH "acute lung injury" OR MH "burn units" OR MH "critical care" OR MH "critical illness" OR MH "intensive care units" OR MH "intubation" OR MH "intubation, intratracheal" OR MH "multiple organ dysfunction syndrome" OR MH "respiratory care units" OR MH "respiration, artificial" OR MH "respiratory distress syndrome, acute" OR MH "respiratory distress syndrome" OR MH "sepsis" OR MH "shock" OR MH "shock, septic" OR MH "ventilators, mechanical" OR MH "bacteremia" OR MH "endotoxemia" OR MH "fungemia" OR MH "candidemia" OR MH "parasitemia" OR MH "viremia" OR "acute lung injuries" OR "acute lung injury" OR "ards" OR "artificial respiration" OR "artificial ventilation" OR "bacteremia" OR "bacteremia" OR "bacteremias" OR "bacteremic" OR "bacteremics" OR "blood poisoning" OR "blood poisonings" OR "burn unit" OR "burn units" OR "candidemia" OR "critical care" OR "critical illness" OR "critical illnesses" OR "critically ill" OR "endotoxemia" OR "endotoxemia" OR "endotoxemias" OR "endotoxemic" OR "endotoxemics" OR "fungemia" OR "i c u " OR "icu" OR "intensive care" OR "multi organ dysfunction" OR "multi organ dysfunctions" OR "multi organ failure" OR "multi organ failures" OR "multi system organ dysfunction" OR "multi system organ dysfunctions" OR "multi system organ failure" OR "multi system organ failures" OR "multiorgan dysfunction" OR "multiorgan dysfunctions" OR "multiorgan failure" OR "multiorgan failures" OR "multiple organ dysfunction" OR "multiple organ dysfunctions" OR "multiple organ failure" OR "multiple organ failures" OR "multisystem organ dysfunction" OR "multisystem organ dysfunctions" OR "multisystem organ failure" OR "multisystem organ failures" OR "pyaemia" OR "pyaemias" OR "pyaemic" OR "pyaemics" OR "pyemia" OR "pyemias" OR "pyemic" OR "pyemics" OR "pyohaemia" OR "pyohaemias" OR "pyohaemic" OR "pyohemia" OR "pyohemias" OR "pyohemic" OR "pyohemics" OR "pyohemics" OR "respiratory distress syndrome" OR "respiratory care unit" OR "respiratory care units" OR "respiratory distress syndromes" OR "sepsis" OR "septic" OR "septicaemia" OR "septicaemias" OR "septicaemic" OR "septicaemics" OR "septicemia" OR "septicemias" OR "shock" OR "viremia" OR "viremias" OR "viremic" OR "viremics" OR bacillaemi* OR bacillemi* OR bacteraemi* OR candidaemi* OR candidemi* OR endotoxaemi* OR fungaemi* OR fungemi* OR intubat* OR "mechanical ventilat*" OR "mechanically ventilat*" OR parasitaemi* OR parasitemi*)

AND

(MH "confusion" OR MH "delirium" OR "agitated emergence" OR "altered consciousness" OR "altered mental state" OR "altered mental states" OR "altered mental status" OR "delire" OR "delirious" OR "delirious" OR "dis orientation" OR "dis orientations" OR "dis oriented" OR "emergence agitation" OR "emergence agitations" OR "emergence excitement" OR "emergence excitements" OR "mental status change" OR "mental status changes" OR "organic brain syndrome" OR "organic brain syndromes" OR "post anaesthetic excitement" OR "post anaesthetic excitements" OR "post anesthetic excitement" OR "post anesthetic excitements" OR "post anaesthesia excitement" OR "post anaesthesia excitements" OR "post anesthesia excitement" OR "post anesthesia excitements" OR "post operative agitation" OR "post operative excitement" OR "post surgical agitation" OR "post surgical excitement" OR "postanaesthetic excitement" OR "postanaesthetic excitements" OR "postanesthetic excitement" OR "postanesthetic excitements" OR "postanaesthesia excitement" OR "postanaesthesia excitements" OR "postanesthesia excitement" OR "postanesthesia excitements" OR "postoperative agitation" OR "postoperative excitement" OR "postsurgical agitation" OR "postsurgical excitement" OR confusion* OR delerium* OR deliria* OR delirium* OR disorient* OR hallucinat* OR illusion* OR illusor*)

AND

(MH "randomized controlled trials" OR MH "clinical trials" OR TI "controlled clinical trial" OR TI "randomized" OR TI "randomised" OR TI "random" OR TI "placebo" OR TI "drug therapy" OR TI "randomly" OR TI "trial" OR TI "groups" OR TI "clinical trial" OR TI "clinical trials" OR AB "controlled clinical trial" OR AB "randomized" OR AB "randomised" OR AB "random" OR AB "placebo" OR AB "drug therapy" OR AB "randomly" OR AB "trial" OR AB "groups" OR AB "clinical trial" OR AB "clinical trials")

481 results

# PsycINFO

( "acute lung injury" OR "burn units" OR "critical care" OR "critical illness" OR "intensive care units" OR "intubation" OR "multiple organ dysfunction syndrome" OR "respiratory care units" OR "respiratory distress syndrome" OR "sepsis" OR "shock" OR "bacteremia" OR "endotoxemia" OR "fungemia" OR "candidemia" OR "parasitemia" OR "viremia" OR "acute lung injuries" OR "acute lung injury" OR "ards" OR "artificial respiration" OR "artificial ventilation" OR "bacteremia" OR "bacteremia" OR "bacteremias" OR "bacteremic" OR "bacteremics" OR "blood poisoning" OR "blood poisonings" OR "burn unit" OR "burn units" OR "candidemia" OR "critical care" OR "critical illness" OR "critical illnesses" OR "critically ill" OR "endotoxemia" OR "endotoxemia" OR "endotoxemias" OR "endotoxemic" OR "endotoxemics" OR "fungemia" OR "i c u " OR "icu" OR "intensive care" OR "multi organ dysfunction" OR "multi organ dysfunctions" OR "multi organ failure" OR "multi organ failures" OR "multi system organ dysfunction" OR "multi system organ dysfunctions" OR "multi system organ failure" OR "multi system organ failures" OR "multiorgan dysfunction" OR "multiorgan dysfunctions" OR "multiorgan failure" OR "multiorgan failures" OR "multiple organ dysfunction" OR "multiple organ dysfunctions" OR "multiple organ failure" OR "multiple organ failures" OR "multisystem organ dysfunction" OR "multisystem organ dysfunctions" OR "multisystem organ failure" OR "multisystem organ failures" OR "pyaemia" OR "pyaemias" OR "pyaemic" OR "pyaemics" OR "pyemia" OR "pyemias" OR "pyemic" OR "pyemics" OR "pyohaemia" OR "pyohaemias" OR "pyohaemic" OR "pyohemia" OR "pyohemias" OR "pyohemic" OR "pyohemics" OR "pyohemics" OR "respiratory distress syndrome" OR "respiratory care unit" OR "respiratory care units" OR "respiratory distress syndromes" OR "sepsis" OR "septic" OR "septicaemia" OR "septicaemias" OR "septicaemic" OR "septicaemics" OR "septicemia" OR "septicemias" OR "shock" OR "viremia" OR "viremias" OR "viremic" OR "viremics" OR bacillaemi* OR bacillemi* OR bacteraemi* OR candidaemi* OR candidemi* OR endotoxaemi* OR fungaemi* OR fungemi* OR intubat* OR "mechanical ventilat*" OR "mechanically ventilat*" OR parasitaemi* OR parasitemi*)

AND

(DE "mental confusion" OR DE "delirium" OR "agitated emergence" OR "altered consciousness" OR "altered mental state" OR "altered mental states" OR "altered mental status" OR "delire" OR "delirious" OR "delirious" OR "dis orientation" OR "dis orientations" OR "dis oriented" OR "emergence agitation" OR "emergence agitations" OR "emergence excitement" OR "emergence excitements" OR "mental status change" OR "mental status changes" OR "organic brain syndrome" OR "organic brain syndromes" OR "post anaesthetic excitement" OR "post anaesthetic excitements" OR "post anesthetic excitement" OR "post anesthetic excitements" OR "post anaesthesia excitement" OR "post anaesthesia excitements" OR "post anesthesia excitement" OR "post anesthesia excitements" OR "post operative agitation" OR "post operative excitement" OR "post surgical agitation" OR "post surgical excitement" OR "postanaesthetic excitement" OR "postanaesthetic excitements" OR "postanesthetic excitement" OR "postanesthetic excitements" OR "postanaesthesia excitement" OR "postanaesthesia excitements" OR "postanesthesia excitement" OR "postanesthesia excitements" OR "postoperative agitation" OR "postoperative excitement" OR "postsurgical agitation" OR "postsurgical excitement" OR confusion* OR delerium* OR deliria* OR delirium* OR disorient* OR hallucinat* OR illusion* OR illusor*)

AND

(DE "randomized controlled trials" OR DE "clinical trials" OR TI "controlled clinical trial" OR TI "randomized" OR TI "randomised" OR TI "random" OR TI "placebo" OR TI "drug therapy" OR TI "randomly" OR TI "trial" OR TI "groups" OR TI "clinical trial" OR TI "clinical trials" OR AB "controlled clinical trial" OR AB "randomized" OR AB "randomised" OR AB "random" OR AB "placebo" OR AB "drug therapy" OR AB "randomly" OR AB "trial" OR AB "groups" OR AB "clinical trial" OR AB "clinical trials")

248 results

# Scopus

TITLE-ABS-KEY("acute lung injury" OR "burn units" OR "critical care" OR "critical illness" OR "intensive care units" OR "intubation" OR "multiple organ dysfunction syndrome" OR "respiratory care units" OR "respiration, artificial" OR "respiratory distress syndrome" OR "sepsis" OR "shock" OR "bacteremia" OR "endotoxemia" OR "fungemia" OR "candidemia" OR "parasitemia" OR "viremia" OR "acute lung injuries" OR "acute lung injury" OR "ards" OR "artificial respiration" OR "artificial ventilation" OR "bacteremia" OR "bacteremia" OR "bacteremias" OR "bacteremic" OR "bacteremics" OR "blood poisoning" OR "blood poisonings" OR "burn unit" OR "burn units" OR "candidemia" OR "critical care" OR "critical illness" OR "critical illnesses" OR "critically ill" OR "endotoxemia" OR "endotoxemia" OR "endotoxemias" OR "endotoxemic" OR "endotoxemics" OR "fungemia" OR "i c u " OR "icu" OR "intensive care" OR "mulorgan dysfunction" OR "mulorgan dysfunctions" OR "mulorgan failure" OR "mulorgan failures" OR "mulsystem organ dysfunction" OR "mulsystem organ dysfunctions" OR "mulsystem organ failure" OR "mulsystem organ failures" OR "multiorgan dysfunction" OR "multiorgan dysfunctions" OR "multiorgan failure" OR "multiorgan failures" OR "multiple organ dysfunction" OR "multiple organ dysfunctions" OR "multiple organ failure" OR "multiple organ failures" OR "multisystem organ dysfunction" OR "multisystem organ dysfunctions" OR "multisystem organ failure" OR "multisystem organ failures" OR "pyaemia" OR "pyaemias" OR "pyaemic" OR "pyaemics" OR "pyemia" OR "pyemias" OR "pyemic" OR "pyemics" OR "pyohaemia" OR "pyohaemias" OR "pyohaemic" OR "pyohemia" OR "pyohemias" OR "pyohemic" OR "pyohemics" OR "pyohemics" OR "respiratory distress syndrome" OR "respiratory care unit" OR "respiratory care units" OR "respiratory distress syndromes" OR "sepsis" OR "septic" OR "septicaemia" OR "septicaemias" OR "septicaemic" OR "septicaemics" OR "septicemia" OR "septicemias" OR "shock" OR "viremia" OR "viremias" OR "viremic" OR "viremics" OR bacillaemi* OR bacillemi* OR bacteraemi* OR candidaemi* OR candidemi* OR endotoxaemi* OR fungaemi* OR fungemi* OR intubat* OR "mechanical ventilat*" OR "mechanically ventilat*" OR parasitaemi* OR parasitemi*)

AND

TITLE-ABS-KEY("confusion" OR "delirium" OR "agitated emergence" OR "altered consciousness" OR "altered mental state" OR "altered mental states" OR "altered mental status" OR "delire" OR "delirious" OR "delirious" OR "dis orientation" OR "dis orientations" OR "dis oriented" OR "emergence agitation" OR "emergence agitations" OR "emergence excitement" OR "emergence excitements" OR "mental status change" OR "mental status changes" OR "organic brain syndrome" OR "organic brain syndromes" OR "post anaesthetic excitement" OR "post anaesthetic excitements" OR "post anesthetic excitement" OR "post anesthetic excitements" OR "post anaesthesia excitement" OR "post anaesthesia excitements" OR "post anesthesia excitement" OR "post anesthesia excitements" OR "post operative agitation" OR "post operative excitement" OR "post surgical agitation" OR "post surgical excitement" OR "postanaesthetic excitement" OR "postanaesthetic excitements" OR "postanesthetic excitement" OR "postanesthetic excitements" OR "postanaesthesia excitement" OR "postanaesthesia excitements" OR "postanesthesia excitement" OR "postanesthesia excitements" OR "postoperative agitation" OR "postoperative excitement" OR "postsurgical agitation" OR "postsurgical excitement" OR confusion* OR delerium* OR deliria* OR delirium* OR disorient* OR hallucinat* OR illusion* OR illusor*)

AND

TITLE-ABS-KEY("randomized controlled trials" OR "clinical trials" OR "controlled clinical trial" OR "randomized" OR "randomised" OR "random" OR "placebo" OR "drug therapy" OR "randomly" OR "trial" OR "groups" OR "clinical trial" OR "clinical trials" OR "controlled clinical trial" OR "randomized" OR "randomised" OR "random" OR "placebo" OR "drug therapy" OR "randomly" OR "trial" OR "groups" OR "clinical trial" OR "clinical trials")

4,272 results

# Web of Science

TS=("acute lung injury" OR "burn units" OR "critical care" OR "critical illness" OR "intensive care units" OR "intubation" OR "multiple organ dysfunction syndrome" OR "respiratory care units" OR "respiration, artificial" OR "respiratory distress syndrome" OR "sepsis" OR "shock" OR "bacteremia" OR "endotoxemia" OR "fungemia" OR "candidemia" OR "parasitemia" OR "viremia" OR "acute lung injuries" OR "acute lung injury" OR "ards" OR "artificial respiration" OR "artificial ventilation" OR "bacteremia" OR "bacteremia" OR "bacteremias" OR "bacteremic" OR "bacteremics" OR "blood poisoning" OR "blood poisonings" OR "burn unit" OR "burn units" OR "candidemia" OR "critical care" OR "critical illness" OR "critical illnesses" OR "critically ill" OR "endotoxemia" OR "endotoxemia" OR "endotoxemias" OR "endotoxemic" OR "endotoxemics" OR "fungemia" OR "i c u " OR "icu" OR "intensive care" OR "mulorgan dysfunction" OR "mulorgan dysfunctions" OR "mulorgan failure" OR "mulorgan failures" OR "mulsystem organ dysfunction" OR "mulsystem organ dysfunctions" OR "mulsystem organ failure" OR "mulsystem organ failures" OR "multiorgan dysfunction" OR "multiorgan dysfunctions" OR "multiorgan failure" OR "multiorgan failures" OR "multiple organ dysfunction" OR "multiple organ dysfunctions" OR "multiple organ failure" OR "multiple organ failures" OR "multisystem organ dysfunction" OR "multisystem organ dysfunctions" OR "multisystem organ failure" OR "multisystem organ failures" OR "pyaemia" OR "pyaemias" OR "pyaemic" OR "pyaemics" OR "pyemia" OR "pyemias" OR "pyemic" OR "pyemics" OR "pyohaemia" OR "pyohaemias" OR "pyohaemic" OR "pyohemia" OR "pyohemias" OR "pyohemic" OR "pyohemics" OR "pyohemics" OR "respiratory distress syndrome" OR "respiratory care unit" OR "respiratory care units" OR "respiratory distress syndromes" OR "sepsis" OR "septic" OR "septicaemia" OR "septicaemias" OR "septicaemic" OR "septicaemics" OR "septicemia" OR "septicemias" OR "shock" OR "viremia" OR "viremias" OR "viremic" OR "viremics" OR bacillaemi* OR bacillemi* OR bacteraemi* OR candidaemi* OR candidemi* OR endotoxaemi* OR fungaemi* OR fungemi* OR intubat* OR "mechanical ventilat*" OR "mechanically ventilat*" OR parasitaemi* OR parasitemi*)

AND

TS=("confusion" OR "delirium" OR "agitated emergence" OR "altered consciousness" OR "altered mental state" OR "altered mental states" OR "altered mental status" OR "delire" OR "delirious" OR "delirious" OR "dis orientation" OR "dis orientations" OR "dis oriented" OR "emergence agitation" OR "emergence agitations" OR "emergence excitement" OR "emergence excitements" OR "mental status change" OR "mental status changes" OR "organic brain syndrome" OR "organic brain syndromes" OR "post anaesthetic excitement" OR "post anaesthetic excitements" OR "post anesthetic excitement" OR "post anesthetic excitements" OR "post anaesthesia excitement" OR "post anaesthesia excitements" OR "post anesthesia excitement" OR "post anesthesia excitements" OR "post operative agitation" OR "post operative excitement" OR "post surgical agitation" OR "post surgical excitement" OR "postanaesthetic excitement" OR "postanaesthetic excitements" OR "postanesthetic excitement" OR "postanesthetic excitements" OR "postanaesthesia excitement" OR "postanaesthesia excitements" OR "postanesthesia excitement" OR "postanesthesia excitements" OR "postoperative agitation" OR "postoperative excitement" OR "postsurgical agitation" OR "postsurgical excitement" OR confusion* OR delerium* OR deliria* OR delirium* OR disorient* OR hallucinat* OR illusion* OR illusor*)

AND

TS=("randomized controlled trials" OR "clinical trials" OR "controlled clinical trial" OR "randomized" OR "randomised" OR "random" OR "placebo" OR "drug therapy" OR "randomly" OR "trial" OR "groups" OR "clinical trial" OR "clinical trials" OR "controlled clinical trial" OR "randomized" OR "randomised" OR "random" OR "placebo" OR "drug therapy" OR "randomly" OR "trial" OR "groups" OR "clinical trial" OR "clinical trials")

1,762 results

# ClinicalTrials.gov

| Delirium/intensive | 220 |
| --- | --- |
| Delirium/ICU | 302 |
| Delirium/critical | 111 |
| Delirium/critically | 77 |
| Altered Mental Status/intensive | 0 |
| Altered Mental Status/ICU | 2 |
| Altered Mental Status/critical | 1 |
| Altered Mental Status/critically | 1 |
| Hallucinations/intensive | 1 |
| Hallucinations/ICU | 1 |
| Hallucinations/critical | 0 |
| Hallucinations/critically | 0 |
| Confusion/intensive | 201 |
| Confusion/ICU | 275 |
| Confusion/critical | 98 |
| Confusion/critically | 69 |
| Postoperative Confusion/intensive | 62 |
| Postoperative Confusion/ICU | 81 |
| Postoperative Confusion/critical | 16 |
| Postoperative Confusion/critically | 4 |
| Postoperative Delirium/intensive | 68 |
| Postoperative Delirium/ICU | 90 |
| Postoperative Delirium/critical | 17 |
| Postoperative Delirium/critically | 4 |
| Total Results | 1,701 |

**Additional File Section 3: Inclusion criteria**

Trials were included in the systematic review if delirium was assessed using a validated screening instrument (1) or diagnostic criteria (2). The validated screening instruments are listed below:

- 3-Minute Diagnostic Confusion Assessment Method (3D-CAM)
- 4AT Rapid Clinical Test for Delirium (4AT)
- Brief Confusion Assessment Method (bCAM)
- Confusion Assessment Method (CAM)
- Confusion Assessment Method for the ICU (CAM-ICU)
- Confusion Assessment Method – Severity (CAM-S)
- Communication Capacity Scale & Agitation-Distress Scale (CCS/ADS)
- Cognitive Test for Delirium (CTD)
- Confusional State Examination (CSE)
- Delirium Index (DI)
- Delirium-O-Meter (DOM)
- Delirium Observation Screening Scale (DOSS)
- Delirium Rating Scale Revised-98 (DRS-R-98)
- Delirium Symptom Interview (DSI)
- Delirium Severity Scale (DSS)
- Family Confusion Assessment Method (FAM-CAM)
- Intensive Care Delirium Screening Checklist (ICDSC)
- Memorial Delirium Assessment Scale (MDAS)
- Neelon-Champagne Confusion Scale (NEECHAM)
- Nursing Delirium Screening Scale (NuDESC)
- Recognizing Acute Delirium as part of Routine (RADAR)
- Recoverable Cognitive Dysfunction Scale (RCDS)

1. Network for Investigation of Delirium: Unifying Scientists (NIDUS). 2018. Delirium Measurement Info Cards [Website]. URL: <https://deliriumnetwork.org/measurement/delirium-info-cards/>
2. Association. AP. Diagnostic and statistical manual of mental disorders: DSM-5. American Psychiatric Association; Washington DC: 2013.

**Additional File Section 4: Delirium outcome categories**

A priori, we defined four categories of delirium outcomes: delirium incidence, delirium composite (separately considered delirium-free days and delirium and coma-free days), delirium duration and delirium severity. In addition to these four categories, we allowed data extractors to populate an “other” field. For each of these four categories plus other, data extractors copied the text from the citation where the outcome was defined.

**Additional File Section 5: Statistical methods categories**

For each delirium outcome, both primary and secondary, that was identified, we extracted the statistical analysis method that was used to compare the delirium outcome across intervention groups. A priori, we defined the statistical methods listed below. In addition, we included an “other” field where data extractors were allowed to provide details of statistical analysis methods that were not included in the a priori defined list. In addition, several statistical methods could have been reported for each delirium outcome:

- Two-sample test for proportion
- Logistic regression model
- Chi-square test
- Fisher’s exact test
- Two-sample t-test
- ANOVA
- Wilcoxon rank-sum test
- Kruskal-Wallis test
- Time to event, Cox PH model
- Time to event, Cox PH model among survivors
- Time to event, Cox PH model, censoring at hospital discharge and death
- Fine and Gray competing risk model (competing risk of death, extubation, ICU discharge, etc)
- Joint model for recurrent event (daily delirium) with competing risk of ICU discharge/death
- Unknown
- Other

**Additional File Table 1: Individual study characteristics of the 65 delirium trials**

| Author (year) | ICU Type(s) | Patient Type(s) | No. Patients | Delirium Screening Instrument | Delirium Outcome(s) | Mortality | ICU LOS |
| --- | --- | --- | --- | --- | --- | --- | --- |
|  |  |  |  |  |  |  |  |
| Abbasi, 2018 | Mixed | Unknown | 137 | CAM-ICU | Incidence, duration | Yes | Yes |
| Abdelgalel, 2016 | Unspecified | MV/ARF | 90 | CAM-ICU | Incidence | Yes | Yes |
| Al-Qadheeb, 2016 | Medical  Surgical | MV/ARF | 68 | ICDSC, DSM Criteria | Incidence, duration | Yes | Yes |
| Alvarez, 2017 | Other^1^ | Other^2^ | 140 | CAM | Incidence, duration, severity | Yes | Yes |
| Anvaripour, 2019 | Surgical | Cardiology | 80 | NEECHAM | Severity | No | No |
| Atalan, 2013 | Unspecified | Cardiology | 53 | CAM-ICU | Duration | Yes | Yes |
| Avidan, 2017 | Unspecified | Surgery | 672 | CAM, CAM-ICU, CAM-S | Incidence, severity | No | No |
| Azeem, 2018 | Unspecified | Cardiology | 60 | CAM-ICU | Incidence | No | Yes |
| Azuma, 2018 | Mixed | MV/ARF | 70 | DSM Criteria, ICDSC | Incidence, duration | No | Yes |
| Bakri, 2015 | Mixed | Surgery | 96 | ICDSC | Duration | No | No |
| Campbell, 2019 | Medical  Surgical  Mixed | Unknown | 200 | CAM-ICU | DCFD, severity | Yes | Yes |
| Damshens, 2018 | Unspecified | Surgery | 80 | CAM-ICU | Incidence | No | Yes |
| Djokic, 2011 | Unspecified | Other^2^ | 240 | DSM Criteria | Severity | Yes | No |
| Dong-Nan Yu, 2017 | Unspecified | Surgery | 92 | CAM, MMSE | Incidence | No | No |
| Eghbali-Babadi, 2017 | Cardiac Surgery | Cardiology | 68 | CAM-ICU | Incidence | No | No |
| Finotto, 2006 | Cardiac Care | Cardiology | 48 | CAM-ICU | Incidence | No | No |
| Gamberini, 2009 | Cardiac Surgery | Surgery | 113 | CAM | Incidence, duration | Yes | Yes |
| Girard, 2010 | Medical  Surgical | MV/ARF | 101 | CAM-ICU | Incidence, DCFD, duration | Yes | Yes |
| Girard, 2018 | Medical  Surgical | MV/ARF, Cardiology, Other^2^ | 566 | CAM-ICU | DCFD, duration | Yes | Yes |
| Guo, 2015 | Surgical | Other^2^ | 156 | CAM-ICU | Incidence | No | No |
| Hakim, 2012 | Cardiac Surgery | Cardiology | 101 | ICDSC, DSM IV | Incidence, duration, severity | Yes | Yes |
| Hasanshahian, 2019 | Unspecified | Unknown | 70 | NEECHAM | Severity | No | No |
| Karadas, 2016 | Medical | MV/ARF | 94 | CAM-ICU | Incidence, duration | No | No |
| Khan, 2018 | Surgical | Surgery | 135 | CAM-ICU | Incidence, duration, severity | No | Yes |
| Khan, 2019 | Medical  Surgical  Other^1^ | MV/ARF, Medical, Surgery | 351 | CAM-ICU | DCFD, severity | Yes | Yes |
| Lei, 2017 | Cardiac Surgery | Cardiology | 249 | CAM-ICU, CAM | Incidence | Yes | Yes |
| Li, 2016 | General | MV/ARF | 44 | CAM-ICU, ICDSC | Incidence | No | Yes |
| Li, 2017 | Unspecified | Surgery | 285 | CAM, CAM-ICU | Incidence, duration | Yes | Yes |
| Liu, 2017 | Surgical | MV/ARF | 105 | CAM-ICU | Incidence, duration | Yes | Yes |
| Luo, 2015 | General  Medical | ARDS | 40 | CAM-ICU | Incidence | Yes | Yes |
| Lyu, 2015 | General | MV/ARF | 140 | CAM-ICU | Incidence, duration | Yes | Yes |
| Ma, 2016 | General | Other | 237 | CAM-ICU | Duration | Yes | Yes |
| Maldonado, 2009 | Unspecified | Cardiology | 118 | DSM Criteria | Incidence, duration | Yes | Yes |
| Mardani, 2012 | Unspecified | Cardiology | 93 | MMSE, DSM Criteria | Incidence | No | Yes |
| Massoumi, 2019 | Unspecified | Cardiology | 88 | CAM-ICU | Incidence, severity | No | Yes |
| Mohammadi, 2016 | Unspecified | Surgery | 40 | CAM-ICU | Incidence, severity | Yes | Yes |
| Moon, 2015 | Unspecified | Unknown | 123 | CAM-ICU | Incidence | Yes | Yes |
| Munro, 2017 | Unspecified | Surgery, Medical | 30 | CAM-ICU, Chart Review | DFD, duration | No | No |
| Needham, 2016 | Unspecified | ARDS, MV/ARF | 272 | CAM-ICU | Incidence | No | Yes |
| Page, 2013 | Mixed | MV/ARF | 141 | CAM-ICU | DCFD | Yes | Yes |
| Page, 2017 | Mixed | MV/ARF | 142 | CAM-ICU | Incidence, DCFD, duration | Yes | Yes |
| Pandharipande, 2007 | Medical  Surgical | MV/ARF | 103 | CAM-ICU | DCFD | Yes | Yes |
| Park, 2014 | Cardiac Surgery | Cardiology | 142 | CAM-ICU | Incidence, duration | No | Yes |
| Peker, 2014 | Medical | MV/ARF | 30 | CAM-ICU | Incidence | No | No |
| Potharajaroen, 2018 | Surgical | Surgery | 61 | CAM-ICU, DSM Criteria | Incidence | No | No |
| Prakanrattana, 2007 | Unspecified | Cardiology | 126 | CAM-ICU | Incidence | No | Yes |
| Robinson, 2014 | Unspecified | Surgery | 301 | CAM-ICU, Chart Review | Incidence, duration | Yes | Yes |
| Rubino, 2010 | Unspecified | Cardiology | 30 | DSM Criteria | Incidence, severity | No | Yes |
| Saager, 2015 | Cardiac Surgery | Cardiology | 198 | CAM, Digit Span | Incidence, severity | No | No |
| Sauer, 2014 | Unspecified | Cardiology | 737 | CAM-ICU, CAM | Incidence, duration | Yes | Yes |
| Shehabi, 2009 | Cardiac Surgery | Cardiology | 299 | CAM-ICU | Incidence, duration | Yes | Yes |
| Shi, 2019 | Unspecified | Cardiology | 164 | CAM | Incidence, duration | No | Yes |
| Simons, 2016 | Mixed | Surgery, Medical, Other | 734 | CAM-ICU, Chart Review | Incidence, DCFD | Yes | Yes |
| Skrobik, 2003 | Mixed | Surgery, Medical | 73 | ICDSC, DSM Criteria | Severity | Yes | No |
| Skrobik, 2018 | Medical  Mixed | ARDS, MV/ARF, Medical, Surgery, Other | 100 | ICDSC | Incidence, DFD, duration | Yes | Yes |
| Strike, 2019 | Cardiac Surgery | Cardiology | 44 | CAM-ICU | Incidence | Yes | Yes |
| Su, 2016 | Unspecified | Surgery | 700 | CAM-ICU | Incidence | Yes | Yes |
| Subramaniam, 2019 | Cardiac Surgery | Cardiology | 120 | DSI, CAM, CAM-ICU | Incidence, duration, severity | No | Yes |
| Taguchi, 2007 | Unspecified | Surgery | 11 | NEECHAM | Incidence; severity | No | No |
| van Eijk, 2010 | Unspecified | Surgery, Medical | 104 | CAM-ICU, CAM, Chart Review | Duration, severity | Yes | Yes |
| Van Rompaey, 2012 | Unspecified | Unknown | 136 | NEECHAM | Incidence | No | No |
| Vijayakumar, 2016 | Unspecified | Other^2^ | 56 | CAM-ICU | Incidence, duration | No | Yes |
| Wang, 2012 | Unspecified | Surgery | 457 | CAM-ICU | Incidence, DFD | Yes | Yes |
| Wang, 2015 | Unspecified | Surgery | 162 | DSM Criteria | Incidence | No | No |
| Whitlock, 2014 | Cardiac Surgery | Cardiology | 310 | CAM-ICU | Incidence | No | Yes |

**Abbreviations.**  *ARF: Acute Respiratory Failure, CAM: Confusion Assessment Method, CAM-ICU: Confusion Assessment Method for the ICU, CAM-S: Confusion Assessment Method – Severity, DSI: Delirium Symptom Interview, DSM: Diagnostic and Statistical Manual of Mental Disorders, ICDSC: Intensive Care Delirium Screening Checklist, ICU: Intensive Care Unit, MMSE:* *Mini-Mental State Examination, MV: Mechanical Ventilation, NEECHAM: Neelon-Champagne Confusion Scale, DFD: Delirium-free days, DCFD: Delirium- and coma-free days*

*^1^ Other ICU Type(s) includes Intermediate Care Unit (Alvarez, 2017 and Khan, 2019)*

^2^ Other Patient Type(s) include elderly non-intubated (Alvarez, 2017), delirium tremens (Djokic, 2011), Shock/need for vasopressors (Girard, 2018), Cancer (Guo, 2015), Organophosphate compound poisoning (Vijayakumar, 2016)

**Additional File Table 2: Individual study risk of bias assessments.**

Legend: Blinding of participants, personnel and outcome assessors and incomplete outcome data were evaluated with respect to the primary delirium outcome.

|  | Random sequence generation (selection bias) | Allocation concealment (selection bias) | Blinding of participants, personnel and outcomes assessors (performance and detection bias) | Incomplete outcome data (attrition bias) | Other Sources of Bias |
| --- | --- | --- | --- | --- | --- |
| Abbasi, 2018 | Low | Low | Low | Unclear | Low |
| Abdelgalel, 2016 | Low | Low | Low | Low | Low |
| Al-Qadheeb, 2016 | Low | Low | Low | Low | Low |
| Alvarez, 2017 | Low | Low | Low | Low | Low |
| Page, 2017 | Low | Low | Low | Unclear | Low |
| Anvaripour, 2019 | Unclear | Unclear | Unclear | Unclear | Unclear |
| Atalan, 2013 | Unclear | Unclear | High | Unclear | Low |
| Avidan, 2017 | Low | Low | Low | Low | Low |
| Azeem, 2018 | Low | Low | High | High | Low |
| Azuma, 2018 | Low | Unclear | High | Low | Low |
| Bakri, 2015 | Low | Unclear | Low | Unclear | Low |
| Campbell, 2019 | Low | Unclear | Low | Low | Low |
| Damshens, 2018 | Unclear | Unclear | High | Low | Low |
| Djokic, 2011 | Low | Unclear | Unclear | Low | Low |
| Dong-Nan Yu, 2017 | Low | Unclear | Unclear | Unclear | Unclear |
| Eghbali-Babadi, 2017 | Low | Unclear | Low | Low | Low |
| Finotto, 2006 | Low | Low | Unclear | High | Unclear |
| Gamberini, 2009 | Low | Low | Low | Low | Low |
| Girard, 2018 | Low | Low | Low | Low | Low |
| Girard, 2010 | Low | Low | Low | Low | Low |
| Guo, 2015 | Unclear | Unclear | Low | Low | Low |
| Hakim, 2012 | Low | Low | Low | Low | Low |
| Hasanshahian, 2019 | Low | High | Low | Low | Low |
| Karadas, 2016 | High | High | Unclear | Low | Low |
| Khan, 2018 | Low | Low | Low | Low | Low |
| Khan, 2019 | Low | Low | Low | Low | Low |
| Lei, 2017 | Low | Unclear | Low | Low | Low |
| Li, 2016 | Low | Low | Low | Unclear | Low |
| Li, 2017 | Low | Unclear | Low | Low | Low |
| Liu, 2017 | Unclear | Unclear | Low | Low | Low |
| Luo, 2015 | Low | Low | Low | Low | Low |
| Lyu, 2015 | Low | Unclear | Unclear | Low | Low |
| Ma, 2016 | Low | Low | Unclear | Low | Low |
| Maldonado, 2009 | Low | Unclear | High | High | High |
| Mardani, 2012 | Unclear | Unclear | Unclear | Low | Low |
| Massoumi, 2019 | Low | Low | Low | Low | Low |
| Mohammadi, 2016 | Unclear | Unclear | Low | Low | Low |
| Moon, 2015 | Low | Low | High | Low | Low |
| Munro, 2017 | Low | Unclear | Unclear | Low | Low |
| Needham, 2016 | Low | Low | Low | Low | Low |
| Page, 2013 | Low | Low | Low | Low | Low |
| Pandharipande, 2007 | Low | Low | Low | Low | Low |
| Park, 2014 | Unclear | Unclear | Unclear | Low | Low |
| Peker, 2014 | Unclear | Low | Unclear | Unclear | Unclear |
| Potharajaroen, 2018 | High | High | Low | Low | Low |
| Prakanrattana, 2007 | Low | Low | Low | Unclear | Low |
| Robinson, 2014 | Low | Low | Low | Unclear | Low |
| Rubino, 2010 | Low | Low | Low | Low | Low |
| Saager, 2015 | Low | Low | Low | High | Low |
| Sauer, 2014 | Low | Low | Low | Low | Low |
| Shehabi, 2009 | Low | Low | Low | Low | Low |
| Shi, 2019 | Low | Unclear | Low | Low | Low |
| Simons, 2016 | Low | Low | High | Unclear | Low |
| Skrobik, 2018 | Low | Low | Low | Unclear | Low |
| Skrobik, 2003 | High | High | Low | Unclear | Low |
| Strike, 2019 | Low | Low | Unclear | Low | Low |
| Su, 2016 | Low | Unclear | Low | Low | Low |
| Subramaniam, 2019 | Low | Low | Low | Low | Low |
| Taguchi, 2007 | Low | Unclear | Unclear | Low | Low |
| van Eijk, 2010 | Low | Low | Low | Unclear | Low |
| Van Rompaey, 2012 | Low | Low | Low | High | Low |
| Vijayakumar, 2016 | Low | Low | Low | Low | Low |
| Wang, 2015 | Low | Unclear | Low | Low | Low |
| Wang, 2012 | Low | Unclear | Low | Low | Low |
| Whitlock, 2014 | Unclear | Unclear | Low | Unclear | Unclear |

**Additional File Table 3: Frequency of primary and secondary delirium outcomes, mortality and ICU length of stay in the 65 delirium trials.**

|  | **Overall** | **Prevention** | **Treatment** | **Both** |
| --- | --- | --- | --- | --- |
|  | **n=65** | **n=44** | **n=12** | **n=9** |
| Delirium incidence |  |  |  |  |
| Primary | 48 (74) | 42 (96) | 0 (0) | 6 (67) |
| Secondary | 8 (12) | 5 (11) | 1 (8) | 2 (22) |
| Reported; not primary or secondary | 0 (0) | 0 (0) | 0 (0) | 0 (0) |
| Delirium composite^1^ |  |  |  |  |
| Primary | 8 (12) | 1 (2) | 4 (33) | 3 (33) |
| Secondary | 5 (8) | 2 (4) | 1 (8) | 2 (22) |
| Reported; not primary or secondary | 0 (0) | 0 (0) | 0 (0) | 0 (0) |
| Delirium duration |  |  |  |  |
| Primary | 6 (9) | 0 (0) | 5 (42) | 1 (11) |
| Secondary | 18 (28) | 14 (32) | 1 (8) | 3 (33) |
| Reported; not primary or secondary | 4 (6) | 4 (9) | 0 (0) | 0 (0) |
| Delirium severity |  |  |  |  |
| Primary | 8 (12) | 2 (5) | 5 (42) | 1 (11) |
| Secondary | 8 (12) | 7 (16) | 1 (8) | 0 (0) |
| Reported; not primary or secondary | 1 (2) | 1 (2) | 0 (0) | 0 (0) |
| Mortality |  |  |  |  |
| Primary^2^ | 1 (2) | 1 (2) | 0 (0) | 0 (0) |
| Secondary | 20 (31) | 9 (21) | 7 (58) | 4 (44) |
| Reported; not primary or secondary | 13 (20) | 10 (23) | 2 (17) | 1 (11) |
| ICU length of stay |  |  |  |  |
| Primary | 0 (0) | 0 (0) | 0 (0) | 0 (0) |
| Secondary | 31 (48) | 18 (41) | 8 (67) | 5 (56) |
| Reported; not primary or secondary | 16 (25) | 13 (30) | 0 (0) | 3 (33) |

Values in the table are count (%)

^1^ Delirium composite outcomes includes delirium-free days and delirium- and coma-free days. Of the 8 trials with a primary delirium composite outcome, 7 are delirium-and coma-free days and 1 is delirium-free days. Among the 5 trials with a secondary delirium composite outcome, 3 are delirium-and coma-free days and 2 are delirium-free days.

^2^ One trial had co-primary endpoints of delirium incidence and mortality.

**Additional File Table 4: Statistical methods applied to delirium incidence, separately for delirium RCTs conducted among critically ill and surgery patients**

| **Statistical method** | **Critically Ill Patients^1^** | | | **Surgery Patients^1^** | | |
| --- | --- | --- | --- | --- | --- | --- |
|  | **Overall** | **Primary Outcome** | **Secondary Outcome** | **Overall** | **Primary Outcome** | **Secondary Outcome** |
|  | n=14 | n=11 | n=3 | n=37 | n=33 | n=4 |
| Two-sample test for proportions^2^ | 13 (93) | 11 (100) | 2 (67) | 34 (92) | 31 (94) | 3 (75) |
| Two-sample test for means^3^ | 0 (0) | 0 (0) | 0 (0) | 1 (3) | 1 (3) | 0 (0) |
| Non-parametric test^4^ | 1 (7) | 0 (0) | 1 (33) | 1 (3) | 1 (3) | 0 (0) |
| Binomial regression model^5^ | 1 (7) | 0 (0) | 1 (33) | 0 (0) | 0 (0) | 0 (0) |
| Longitudinal regression model^6^ | 1 (7) | 0 (0) | 1 (33) | 1 (3) | 1 (3) | 0 (0) |
| Survival analysis^7^ | 3 (21) | 2 (18) | 1 (33) | 6 (16) | 4 (12) | 2 (50) |
| Competing risk survival analysis^8^ | 0 (0) | 0 (0) | 0 (0) | 1 (3) | 1 (3) | 0 (0) |
| Joint model^9^ | 1 (7) | 1 (9) | 0 (0) | 0 (0) | 0 (0) | 0 (0) |

Values in the table are count (%) of trials evaluating delirium incidence as a primary or secondary outcome. Several statistical methods may be reported for each outcome, therefore column counts (%s) will not sum to the number of primary or secondary outcomes or 100 percent.

^1^ Of the 65 RCTs, 17 and 41 included critically ill and surgery patients, respectively. The remaining 18 RCTs were conducted among other patient populations. A primary or secondary delirium incidence outcome was reported in 14 of 17 and 37 of 41 RCTs including critically ill and surgery patients, respectively. One RCT named delirium incidence as a primary or secondary outcome included both critically ill and surgery patients; this trial is counted in both subgroups.

^2^ Two-sample test for proportions includes two-sample test for proportions assuming normally distributed sample proportions, Fisher’s exact test, Chi-square test, and logistic regression model

^3^ Two-sample test for means includes two-sample t-test, analysis of variance or linear regression model

^4^ Non-parametric test for continuous or ordinal outcomes includes Mann-Whitney test, Wilcoxon rank-sum test, Kruskal Wallis test, and the proportional odds logistic regression model

^5^ Binomial regression model defines the number of days with delirium as the Binomial outcome and the number of days in the ICU as the offset/denominator.

^6^ Longitudinal regression model includes marginal longitudinal logistic regression models for daily delirium and random effects logistic regression models for daily delirium

^7^ Survival analysis defined the outcome as time from randomization to delirium onset with patients censored at ICU discharge or death; statistical comparisons were made using the log-rank test or the Cox proportional hazards regression model

^8^ Competing risk survival analysis defined the outcome as time from randomization to delirium onset with i) patients censored at ICU discharge and death defined as a competing risk or ii) ICU discharge and death defined as competing risks; statistical comparisons were made using the Fine and Gray competing risk model

^9^ Joint model refers to the joint model for recurrent event outcomes (e.g. recurrent delirium events) with terminating event (e.g. ICU discharge or death) proposed by Rondeau (24)

**Additional File Table 5: Statistical methods applied to the delirium composite, for all trials with a delirium composite outcome and separately for trials conducted among critically ill and surgery patients.**

|  | **All Trials** | | | **Critically Ill Patients^2^** | | | **Surgery Patients^2^** | | |
| --- | --- | --- | --- | --- | --- | --- | --- | --- | --- |
|  | Overall^1^ | Primary^1^ | Secondary^1^ | Overall | Primary | Secondary | Overall | Primary | Secondary |
|  | n=13 | n=8 | n=5 | n=8 | n=6 | n=2 | n=6 | n=3 | n=3 |
| Two-sample test for proportions^3^ | 1 (8) | 1 (13) | 0 (0) | 0 (0) | 0 (0) | 0 (0) | 1 (17) | 1 (33) | 0 (0) |
| Two-sample test for means^4^ | 4 (31) | 2 (25) | 2 (40) | 3 (38) | 2 (33) | 1 (50) | 1 (17) | 0 (0) | 1 (33) |
| Non-parametric test^5^ | 11 (85) | 6 (75) | 5 (100) | 7 (88) | 5 (83) | 2 (100) | 5 (83) | 2 (67) | 3 (100) |
| Poisson regression model^6^ | 2 (15) | 1 (13) | 1 (20) | 0 (0) | 0 (0) | 0 (0) | 0 (0) | 0 (0) | 0 (0) |
| Joint model^7^ | 1 (8) | 1 (13) | 0 (0) | 1 (12) | 1 (17) | 0 (0) | 0 (0) | 0 (0) | 0 (0) |

Values in the table are count (%). Several statistical methods may be reported for each outcome, therefore column counts (%s) will not sum to the number of primary or secondary outcomes or 100 percent.

^1^ The sample size, n, reported as “overall” is the total number of delirium composite outcomes, both primary and secondary, whereas the sample size reported for primary and secondary delirium composite outcomes is the number of trials. A trial may report multiple delirium composite outcomes; e.g. delirium- and coma-free days by 14 or 28 days as the primary and secondary outcomes, respectively. A total of 13 delirium composite outcomes were reported by 11 of the 65 trials; 6, 2 and 3 trials reported only a primary, both a primary and secondary or only a secondary delirium composite outcome, respectively.

^2^ Of the 65 RCTs, 17 and 41 included critically ill and surgery patients, respectively. The remaining 18 RCTs were conducted among other patient populations. A primary or secondary delirium composite outcome was reported in 8 of 17 and 6 of 41 RCTs including critically ill and surgery patients, respectively. Three RCTs with a delirium composite as a primary or secondary outcome included both critically ill and surgery patients; these trials are counted in both subgroups.

^3^ Two-sample test for proportion refers to a Fisher’s exact test comparing the distribution of delirium- and coma-free days across treatment groups

^4^ Two-sample test for means includes two-sample t-test, analysis of variance or linear regression model

^5^ Non-parametric test for continuous or ordinal outcomes includes Mann-Whitney test, Wilcoxon rank-sum test, Kruskal Wallis test, and the proportional odds logistic regression model

^6^ Poisson regression model defines the number of days with delirium as the Poisson outcome and the number of days in the ICU as the offset.

^7^ Joint model refers to the joint model for recurrent event outcomes (e.g. recurrent delirium events) with terminating event (e.g. ICU discharge or death) proposed by Rondeau (24)

**Additional File Table 6: Statistical methods applied to delirium duration, for all trials with a delirium duration outcome and separately for trials conducted among critically ill and surgery patients.**

| **Statistical method** | **All Trials** | | | **Critically Ill Patients^1^** | | | **Surgery Patients^1^** | | |
| --- | --- | --- | --- | --- | --- | --- | --- | --- | --- |
|  | Overall | Primary | Secondary | Overall | Primary | Secondary | Overall | Primary | Secondary |
|  | n=24 | n=6 | n=18 | n=8 | n=0 | n=8 | n=14 | n=3 | n=11 |
| Two-sample test for proportions^2^ | 1 (4) | 1(17) | 0 (0) | 0 (0) | 0 (0) | 0 (0) | 1 (7) | 1 (33) | 0 (0) |
| Two-sample test for means^3^ | 9 (38) | 2 (33) | 7 (33) | 3 (37) | 0 (0) | 3 (37) | 4 (28) | 1 (33) | 3 (27) |
| Non-parametric test^4^ | 12 (50) | 2 (33) | 10 (56) | 5 (63) | 0 (0) | 5 (63) | 9 (64) | 2 (67) | 7 (64) |
| Poisson regression model^5^ | 2 (8) | 1 (17) | 1 (6) | 0 (0) | 0 (0) | 0 (0) | 1 (7) | 0 (0) | 1 (9) |
| Survival analysis^6^ | 3 (13) | 2 (33) | 1 (6) | 0 (0) | 0 (0) | 0 (0) | 2 (14) | 1 (33) | 1 (9) |

Values in the table are count (%) of trials with delirium duration as an outcome. Several statistical methods may be reported for each outcome, therefore column counts (%s) will not sum to the number of primary or secondary outcomes or 100 percent.

^1^ Of the 65 RCTs, 17 and 41 included critically ill and surgery patients, respectively. The remaining 18 RCTs were conducted among other patient populations. A primary or secondary delirium duration outcome was reported in 8 of 17 and 14 of 41 RCTs including critically ill and surgery patients, respectively. Two trials with delirium duration as a primary or secondary outcome included both critically ill and surgery patients; these trials are counted in both subgroups

^2^ Two-sample test for proportion refers to a Fisher’s exact test comparing the distribution of days with delirium across treatment groups

^3^ Two-sample test for means includes two-sample t-test, analysis of variance or linear regression model

^4^ Non-parametric test for continuous or ordinal outcomes includes Mann-Whitney test, Wilcoxon rank-sum test, Kruskal Wallis test, and the proportional odds logistic regression model

^5^ Poisson regression model defines the number of days with delirium as the Poisson outcome and the number of days in the ICU as the offset.

^6^ Survival analysis defined the outcome as time from randomization to delirium resolution with patients censored at ICU discharge or death; statistical comparisons were made using the log-rank test or the Cox proportional hazards regression model

**Additional File Table 7: Statistical methods applied to delirium severity, for all trials with a delirium severity outcome and separately for trials conducted among critically ill and surgery patients.**

| **Statistical method** | **All Trials^1^** | | | **Critically Ill Patients^1^** | | | **Surgery Patients^1^** | | |
| --- | --- | --- | --- | --- | --- | --- | --- | --- | --- |
|  | Overall | Primary | Secondary | Overall | Primary | Secondary | Overall | Primary | Secondary |
|  | n=16 | n=8 | n=8 | n=1 | n=1 | n=0 | n=11 | n=3 | n=8 |
| Two-sample test for means^2^ | 3 (19) | 1 (13) | 2 (25) | 0 (0) | 0 (0) | 0 (0) | 2 (18) | 0 (0) | 2 (25) |
| Non-parametric test^3^ | 10 (63) | 4 (50) | 6 (75) | 0 (0) | 0 (0) | 0 (0) | 8 (73) | 2 (67) | 6 (75) |
| Longitudinal regression model^4^ | 5 (31) | 3 (38) | 2 (25) | 1 (100) | 1 (100) | 0 (0) | 3 (27) | 1 (33) | 2 (25) |

Values in the table are count (%) of trials with delirium severity as an outcome. Several statistical methods may be reported for each outcome, therefore column counts (%s) will not sum to the number of primary or secondary outcomes or 100 percent.

^1^ A primary or secondary delirium severity outcome was reported in 16 of 65 delirium RCTs. The 16 RCTs included 10 conducted on surgery patients, 1 including both critically ill and surgery patients and 5 conducted among other patient populations. The 1 RCT including both critically ill and surgery patients is counted in both subgroups.

^2^ Two-sample test for means includes two-sample t-test, analysis of variance or linear regression model

^3^ Non-parametric test for continuous or ordinal outcomes includes Mann-Whitney test, Wilcoxon rank-sum test, Kruskal Wallis test, and the proportional odds logistic regression model

^4^ Longitudinal regression model includes marginal longitudinal linear regression models and random effects linear regression models for delirium severity.

**Additional File Figure 1: Risk of bias analysis**

Legend: The figure displays the proportion of the 65 delirium trials considered to have low, high or unclear risk of bias. Blinding of participants, personnel and outcome assessors and incomplete outcome data were evaluated with respect to the primary delirium outcome.
